# Health Care Setting and Minimally Adequate Depression Treatment Among Publicly Insured Children

**DOI:** 10.1001/jamanetworkopen.2025.28345

**Published:** 2025-08-28

**Authors:** Janet R. Cummings, Xin Hu, Ilana Graetz, Jordan Marchak, Cristian Ramos, Xu Ji

**Affiliations:** 1 Department of Health Policy and Management, Emory University Rollins School of Public Health, Atlanta, Georgia; 2 Department of Radiation Oncology, Emory University School of Medicine, Atlanta, Georgia; 3 Department of Pediatrics, Emory University School of Medicine, Atlanta, Georgia; 4 Aflac Cancer & Blood Disorders Center, Children’s Healthcare of Atlanta, Atlanta, Georgia

## Abstract

**Importance:**

The prevalence of depression among children and adolescents has increased, yet many do not receive adequate treatment. The rise of telemental health care highlights the need to examine how delivery setting changes are associated with depression care.

**Objective:**

To estimate the association between service delivery setting (telehealth vs clinic-only care) and the receipt of mental health treatment among publicly insured children and adolescents with depression.

**Design, Setting, and Participants:**

This cross-sectional study of publicly insured children and adolescents (ages 3-17 years) with an index depression diagnosis used multistate claims data from 41 states and Washington, District of Columbia, to examine the association between health service setting and minimally adequate depression care in 2016 to 2019 (before COVID-19) and 2020. Data were analyzed from June 14, 2022, to March 15, 2024.

**Exposure:**

Mental health service setting categories included most mental health services via telehealth (>50%), some mental health services via telehealth (≤50%), any in-home visits (no telehealth), any in-school visits (no telehealth or in-home visits), and clinic-only visits (reference group).

**Main Outcomes and Measures:**

Outcomes of interest were dichotomous measures of at least 4 mental health visits within 12 weeks of index diagnosis, minimally adequate pharmacotherapy (≥84 days of antidepressant medication fills within 144 days of index diagnosis), and minimally adequate treatment (≥4 visits or minimally adequate pharmacotherapy). Multiple logistic regressions controlled for individual- and contextual-level covariates, state, and year of index diagnosis (2016-2019 vs 2020).

**Results:**

Among 799 452 publicly insured children with an index depression diagnosis from 2016 to 2020, most were aged 12 to 17 years (640 738 [80.1%]) and female (469 014 [58.7%]). The percentage with any telemental health visit increased from 4.5% before the COVID-19 pandemic to 49.8% in 2020, while clinic-only care declined from 76.8% before COVID-19 to 43.1% in 2020. In 2020, children receiving some or most mental health services via telehealth were more likely to receive minimally adequate depression treatment than those receiving clinic-only services (some telehealth: 50.9%; mostly telehealth: 54.5%; clinic-only: 32.1%; *P* < .001 for both comparisons). Differences remained significant in regressions (some telehealth: marginal effect [ME], 16.1 [95% CI, 14.6-17.6] percentage points; mostly telehealth: ME, 19.8 [95% CI, 18.2-21.5] percentage points).

**Conclusions and Relevance:**

In this cross-sectional study of publicly insured children and adolescents, receipt of telemental health services in 2020 was positively associated with minimally adequate depression care. These findings underscore the importance of continued telemental health coverage use by state Medicaid programs and Children’s Health Insurance Programs to facilitate access and minimize barriers to mental health care.

## Key Points

**Question:**

How did care settings change among publicly-insured children with a depression diagnosis in 2020 compared with 2016 to 2019, and were changes associated with receipt of minimally adequate care?

**Findings:**

This cross-sectional study using claims data from 799 452 publicly insured children with depression in 41 states and Washington, District of Columbia, found that use of telemental health care increased in 2020 compared with 2016 to 2019. Children who received telemental health services in 2020 were more likely to receive minimally adequate treatment than those who received services in clinic settings only.

**Meaning:**

The findings of this study support continued telemental health coverage by public insurance programs to ensure access to mental health care.

## Teaser

This cross-sectional study estimates rates and associations of telemental health care with receipt of depression treatment among publicly insured children and adolescents.

## Introduction

Depression is one of the most common mental health disorders among children and adolescents, and its prevalence has increased dramatically.^1,2^ For example, the prevalence of major depression among adolescents in the past year increased from 8.1% in 2009 to 19.5% in 2022.^3,4^ Moreover, children whose families live in poverty are at increased risk for depression due to increased exposure to traumatic events,^5^ family disruption,^6^ and reduced parental involvement.^7^

Public insurance programs, including Medicaid and the Children’s Health Insurance Program (CHIP), provided coverage to more than 36 million children during each month of 2024,^8,9^ including those from families with low income, with disabilities, and/or living in foster care. Although effective treatments (including psychosocial services and/or antidepressant medication) are available for children with depression,^10,11,12^ many do not receive any mental health care.^13^ Among Medicaid-enrolled children with depression who initiate treatment, rates of treatment discontinuity and dropout are high.^13^

Prior studies examining care delivered to Medicaid-enrolled children have typically focused on services offered in clinic-based settings.^13,14,15^ However, barriers exist to children receiving consistent services in clinic-based settings, including distance to the nearest Medicaid-accepting health care practitioner,^16,17,18^ transportation availability,^16,19^ and family scheduling conflicts.^19,20,21,22^ Service delivery models are changing to provide more options for children to receive services outside of clinics, especially with the rapid uptake of telemental health care since 2020.^23^ Prior to the COVID-19 pandemic, telemental health care availability and coverage was extremely limited in many Medicaid and CHIP programs and often excluded services delivered to the home.^24^ Additionally, there have increases in community-based service delivery in school^25,26^ and home settings,^27^ which also reduce geographic barriers to care. In a 2022 study, Medicaid-enrolled children with depression who received in-home services (vs those who did not) were significantly more likely to receive at least 4 psychosocial service visits in the first 12 weeks of treatment and had a longer treatment episode duration.^27^

To date, there is little information about whether service delivery in other settings that reduce geographic barriers, including telehealth or school settings, is associated with changes in mental health care received among publicly insured children with depression. Using multistate claims data, we examined how health care settings were associated with the receipt of mental health care among publicly insured children and adolescents with depression before and after the onset of the COVID-19 pandemic.

## Methods

This cross-sectional study received expedited approval from the Emory University institutional review board, with a waiver for the requirement to obtain informed consent because the study used secondary data, posed minimal risk to participants, and could not be practicably carried out without the waiver, as the study team was unable to contact participants if consent had been required. This study is reported following the Strengthening the Reporting of Observational Studies in Epidemiology (STROBE) reporting guideline for cross-sectional studies.

### Data Sources

Medicaid and CHIP claims data were extracted from the 2016 to 2020 Transformed Medicaid Statistical Information System Analytic Files in the Virtual Research Data Center (VRDC).^28,29^ Using geographic identifiers (zip code, county, state), we merged area-level measures from the area health resources files,^30^ the Social Deprivation Index county files,^31^ and county-level broadband internet connection files.^32^

### Analytic Sample

We derived a sample of publicly insured children and adolescents (ages 3-17 years) with an index depression diagnosis in 2016 to 2020 using the *International Statistical Classification of Diseases and Related Health Problems, Tenth Revision (ICD-10)* codes listed in eTable 1 in Supplement 1 on at least 1 inpatient or 2 outpatient health care claims during the study period. To identify the index diagnosis, we required a 90-day period preceding the index visit (ie, a lookback window) without any depression diagnosis, any mental health visit identified with relevant procedure codes (eTable 2 in Supplement 1), and/or any filled antidepressant medication (eTable 3 in Supplement 1) identified with relevant National Drug Codes.^13,18,27^ We further required continuous Medicaid/CHIP enrollment during the 270 days prior to the index date until 144 days after the index visit, allowing for an administrative enrollment gap of 30 days. Continuous enrollment ensures a sample with stable insurance coverage for the year when each outcome is assessed and facilitates the capture health services utilization for the lookback window and outcome assessment. For children with multiple depression episodes identified, we only included their first episode. Through this process, we identified 1 188 764 children with an index depression diagnosis during the study period (eFigure in Supplement 1).

From this sample, we excluded 83 879 children for whom any part of their episode window (index visit to 144 days after) and the 90-day lookback window occurred outside of the 2016 to 2020 timeframe. To be able to determine service delivery setting for outpatient care, we also excluded children without any outpatient visits that had a depression diagnosis (eTable 1 in Supplement 1) or a mental health–related procedure code (eTable 2 in Supplement 1) in the 144-day period following the index visit. Next, we excluded 47 828 children who had Medicaid records from multiple states and 48 690 children who were missing information on demographic and geographic characteristics. Finally, we excluded 195 522 children living in 9 states that were classified as having high data quality concerns on the primary files used (ie, other service files and pharmacy files).^33^ Our primary analytic sample included 799 452 children from 41 states and Washington, District of Columbia (DC), and we conducted sensitivity analyses with a sample of 994 974 children from all 50 states and Washington, DC. The sample derivation process is presented in eFigure in Supplement 1.

### Outcome Measures

Clinical guidelines have stated that depression among children and adolescents can be treated with psychosocial services and/or antidepressant medication, depending on the severity and presentation of symptoms.^10,11,12^ Treatment includes an acute phase (with a goal to achieve response and ultimately remission) and a continuation phase (to secure response and avoid relapse). Drawing on clinical guidelines and prior literature,^10,11,12,13,14,18^ we created 5 dichotomous variables to measure depression treatment and receipt of minimally adequate care in the acute phase. We used 2 dichotomous indicators to assess treatment initiation: (1) any mental health visit for those with at least 1 outpatient health care claim with a mental health–related Current Procedural Terminology code, including psychotherapy, psychosocial services, case management, or other mental health services (eTable 2 in Supplement 1) within 12 weeks (ie, 84 days) of the index diagnosis, and (2) any pharmacotherapy for individuals who filled an antidepressant prescription during the 144 days following their index visit (eTable 3 in Supplement 1). Next, an indicator was created for those who received at least 4 mental health visits within 12 weeks of the index diagnosis. A 4-visit threshold has been used in prior studies,^13,14,18^ and evidence from the literature suggests that brief interventions with children can lead to reductions in depression symptoms within 4 sessions.^11,34^ Fourth, consistent with prior studies,^13,14,18^ an indicator for minimally adequate pharmacotherapy assessed whether a child had antidepressant prescription fills that covered at least 84 days of the 144 days following their index depression diagnosis. A fifth measure of minimally adequate care identified individuals who received at least 4 mental health visits and/or minimally adequate pharmacotherapy. Notably, this final indicator is meant to capture a minimum, conservative threshold of mental health care that should be received by any child with depression rather than the level of care needed for a full course of treatment.

### Health Care Setting

We used data from the other service files to measure the service delivery setting for mental health care. Specifically, we extracted all mental health–related visits, defined as outpatient health care claims with a mental health diagnosis (eTable 1 in Supplement 1) or with a mental health–related procedure code (eTable 2 in Supplement 1) during the first 12 weeks (84-day) window following the index diagnosis. We used the place of service code on each claim to classify service delivery setting, which provides information about where the service was performed. We identified services delivered through telehealth using the place of service code (02), modifiers (GT, GQ, or 95), and/or telehealth-specific procedure codes (T1014, Q3014, G0425-G0427, G0406-G0408, G0459, G0508, G0509, 0188T, 0189T, 98966-98968, 99441-99444, 99421-99423, G2061-G2063).^35,36^ Services delivered at the child’s home (ie, “Location other than a hospital or other facility, where the patient receives care in a private residence”^37^) were identified using place of service code 12. Services delivered at the child’s school (ie, “a facility whose primary purpose is education”^37^) were identified using place of service code 03. The remaining claims were classified as clinic setting.

Because prior research reported major shifts to telemental health services in 2020 for publicly insured children^23,38^ and because telemental health services reduce logistical barriers to care for this population,^39^ the association between service delivery via telehealth and the receipt of depression care was a focus of this study. Thus, consistent with prior research examining a service delivery setting of interest,^18^ we implemented priority coding to create a categorical variable of service delivery setting with 5 mutually exclusive groups. We first identified children who received some, but not most (≤50%), mental health visits via telehealth (ie, some telemental health visits) and most mental health visits (>50%) via telehealth. Children who did not receive any telemental health visits were then classified into the remaining 3 groups: at least 1 in-home mental health visit, but no telemental health visits; at least 1 in-school mental health visit, but no in-home or telemental health visits; and visits only in the clinic setting (ie, clinic only).

### Covariates

Covariates were informed by the behavioral model of health services use by Andersen and Davidson.^40^ Individual-level covariates included predisposing (ie, age, sex, race and ethnicity), enabling (ie, Medicaid health plan type and eligibility category at index visit), and need-related characteristics (ie, coexisting mental health conditions). Race and ethnicity were self-reported and categorized as Hispanic, non-Hispanic American Indian or Alaskan Native, non-Hispanic Asian or Pacific Islander, non-Hispanic Black, non-Hispanic multiracial group or unknown, and non-Hispanic White.^41^ Six indicators for coexisting mental health conditions were identified in the 144-day observation period following index diagnosis, using at least 1 inpatient or 2 outpatient claims associated with the appropriate diagnosis codes (eTable 1 in Supplement 1).

At the county level, we included several covariates that that were associated with the receipt of telemental health services among Medicaid-enrolled children in prior research.^38^ These include the percentage of Black residents (quartiles), percentage of Hispanic residents (quartiles), Social Deprivation Index (quartiles), and metropolitan status. We also included an additional measure of county-level infrastructure that may be correlated with the receipt of telemental health services: the percentage of households with broadband connections with downstream speed at least 10 megabits per second.^42^

### Statistical Analysis

Given the change in health care delivery landscape and telehealth policy changes during the COVID-19 public health emergency,^43,44,45^ the associations between health care setting and depression treatment receipt may differ in 2016 to 2019 vs 2020. Thus, we examine health care setting for these 2 time periods. In bivariate analyses, comparisons for each outcome in a given setting across time (2020 vs 2016-2019) were conducted with χ^2^ tests. In regression analyses, we used generalized estimating equations with a binomial distribution and logit link function to examine the association between health care setting and each outcome, adjusting for covariates, state of residence, and an indicator for year of index diagnosis (2016-2019 vs 2020). To facilitate interpretation in terms of probability changes of the study outcome associated with care settings and study covariates,^46^ we converted odds ratios into marginal effects (MEs) using the margins macro program^47^ in SAS statistical software version 7.1 (SAS Institute). MEs were estimated as average MEs, in which predicted probabilities were first calculated for each individual based on their observed covariate values, and then we calculated the mean across the sample rather than fixing covariates at their sample means. Furthermore, we interacted health care setting and a pre-post (2016-2019 vs 2020) indicator in the regression models, generating separate MEs for each period. Standard errors were clustered at the county level. All data analysis were conducted using SAS Enterprise Guide version 7.1 (SAS Institute) through the VRDC.^48^ Statistical significance was determined using 2-sided tests and *P* < .05. Data were analyzed from June 14, 2022, to March 15, 2024.

## Results

### Sample Characteristics

Of 799 452 children in our sample with an index depression diagnosis, 640 738 (80.1%) were adolescents aged 12 to 17 years, 469 014 (58.7%) were female, and 641 155 (80.2%) were enrolled in a comprehensive managed care plan (Table 1). Sample characteristics by setting and time period are presented in eTable 4 in Supplement 1.

**Table 1.** Characteristics of Included Publicly Insured Children With Depression

Characteristic	Children, No. (column %)		
	Total (N = 799 452)	2016-2019 (n = 701 327)	2020 (n = 98 125)
Outcome measures			
Any mental health visit^a^	542 705 (67.9)	474 985 (67.7)	67 720 (69.0)
≥4 Mental health visits^a^	243 332 (30.4)	211 976 (30.2)	31 356 (32.0)
Any pharmacotherapy^b^	246 541 (30.8)	215 871 (30.8)	30 670 (31.3)
Minimally adequate pharmacotherapy^c^	110 560 (13.8)	95 448 (13.6)	15 112 (15.4)
Minimally adequate treatment^d^	320 896 (40.1)	279 110 (39.8)	41 786 (42.6)
Individual-level covariates			
Age group, y			
3-11	158 714 (19.9)	141 437 (20.2)	17 277 (17.6)
12-17	640 738 (80.1)	559 890 (79.8)	80 848 (82.4)
Sex			
Female	469 014 (58.7)	408 941 (58.3)	60 073 (61.2)
Male	330 438 (41.3)	292 386 (41.7)	38 052 (38.8)
Race and ethnicity^e^			
Hispanic	217 004 (27.1)	191 746 (27.3)	25 258 (25.7)
Non-Hispanic American Indian or Alaskan Native	16 515 (2.1)	14 733 (2.1)	1782 (1.8)
Non-Hispanic Asian or Pacific Islander	14 755 (1.8)	13 102 (1.9)	1653 (1.7)
Non-Hispanic Black	115 793 (14.5)	101 874 (14.5)	13 919 (14.2)
Non-Hispanic multiracial group or unknown	142 251 (17.8)	125 547 (17.9)	16 704 (17.0)
Non-Hispanic White	293 134 (36.7)	254 325 (36.3)	38 809 (39.6)
Medicaid eligibility type^f^			
Low income	722 190 (90.3)	631 464 (90.0)	90 726 (92.5)
Disability	55 712 (7.0)	50 077 (7.1)	5635 (5.7)
Other or unknown	21 550 (2.7)	19 786 (2.8)	1764 (1.8)
Plan type^g^			
Comprehensive managed care organization	641 155 (80.2)	560 280 (79.9)	80 875 (82.4)
Primary care case management	30 259 (3.8)	26 550 (3.8)	3709 (3.8)
Prepaid health plan	75 947 (9.5)	66 580 (9.5)	9367 (9.5)
Other or unknown	52 091 (6.5)	47 917 (6.8)	4174 (4.3)
Coexisting conditions			
Attention deficit hyperactivity disorder	103 560 (13.0)	91 468 (13.0)	12 092 (12.3)
Anxiety disorder	142 011 (17.8)	120 664 (17.2)	21 347 (21.8)
Autism	10 039 (1.3)	8566 (1.2)	1473 (1.5)
Disruptive, impulse control, and conduct disorders	52 066 (6.5)	46 424 (6.6)	5642 (5.7)
Trauma and other stressor-related disorders	88 300 (11.0)	76 130 (10.9)	12 170 (12.4)
Other mental health conditions	75 343 (9.4)	67 791 (9.7)	7552 (7.7)
County-level covariates			
% Non-Hispanic Black population, quartile^h^			
1	60 285 (7.5)	53 089 (7.6)	7196 (7.3)
2	131 932 (16.5)	115 326 (16.4)	16 606 (16.9)
3	307 917 (38.5)	268 990 (38.4)	38 927 (39.7)
4	299 318 (37.4)	263 922 (37.6)	35 396 (36.1)
% Hispanic population, quartile^h^			
1	71 107 (8.9)	63 557 (9.1)	7550 (7.7)
2	98 627 (12.3)	86 305 (12.3)	12 322 (12.6)
3	185 700 (23.2)	162 819 (23.2)	22 881 (23.3)
4	444 018 (55.5)	388 646 (55.4)	55 372 (56.4)
Social Deprivation Index, quartiles^i^			
1	97 463 (12.2)	477 285 (12.2)	12 151 (12.4)
2	161 286 (20.2)	141 770 (20.2)	19 516 (19.9)
3	212 921 (26.6)	187 936 (26.8)	24 985 (25.5)
4	327 782 (41.0)	286 309 (40.8)	41 473 (42.3)
Households with broadband connections with downstream speed ≥10 mbps, %^j^			
0-40	97 677 (12.2)	90 253 (12.9)	7424 (7.6)
40.1-60	215 121 (26.9)	192 668 (27.5)	22 453 (22.9)
60.1-80	421 007 (52.7)	366 074 (52.2)	54 933 (56.0)
80.1-100	65 647 (8.2)	52 332 (7.5)	13 315 (13.6)
Metropolitan status^k^			
Metropolitan	651 661 (81.5)	571 992 (81.5)	79 669 (81.2)
Nonmetropolitan urban	132 815 (16.6)	116 227 (16.6)	16 588 (16.9)
Rural	14 976 (1.9)	13 108 (1.9)	1868 (1.9)

^a^ Measured in the 12 weeks following the index diagnosis.

^b^ Measured as any antidepressant medication filled in the 144 days following the index diagnosis.

^c^ Measured as a fill or refill for an antidepressant medication for 84 of the 144 days following the index diagnosis.

^d^ Measured as whether the child received at least 4 mental health visits in 12 weeks following index diagnosis or minimally adequate pharmacotherapy.

^e^ Applicants are asked to self-report their race and ethnicity; however, there is variation in the data collection procedures across state Medicaid programs and Children’s Health Insurance Programs. The Centers for Medicare & Medicaid Services provided technical guidance to states on submitting race/ethnicity data to the T-MSIS system.^41^

^f^ Eligibility information extracted in the month of episode initiation.

^g^ Plan type information extracted in the month of episode initiation.

^h^ Information extracted from 2020 and 2021 Area Health Resources Files.^30^ Year-specific county-level measures were linked for individuals identified in 2016-2020.

^i^ Social Deprivation Index generated based on American Community Survey data.^31^ Year-specific data were available from 2016 to 2019. Year-specific data were linked for individuals identified in 2016 to 2019. Data from 2019 were used to link with individuals identified in 2020.

^j^ Broadband information available from 2016 to 2019.^32^ Year-specific broadband information was linked for individuals identified in 2016 to 2019. Broadband information from 2019 was used to link with individuals identified in 2020.

^k^ Defined based on 2013 Rural-Urban Continuum Codes. Metropolitan includes codes 1, 2, and 3; nonmetropolitan urban, 4, 5, 6, and 7; and rural, 8 and 9.

The annual number of children with an index depression diagnosis was greater during the pre–COVID-19 period (mean per year, 175 332 children; 701 327 children across 2016-2019) than in 2020 (98 125 children) (Table 1). Among children with an index depression diagnosis, the percentage with some telemental health visits increased from 4.1% in 2016 to 2019 to 27.3% in 2020, and the percentage of children who received the most mental health visits via telehealth increased from 0.4% in 2016 to 2019 to 22.5% in 2020 (Figure). The receipt of services in other settings declined (in-clinic only services: 76.8% to 43.1%; any in-home services [no telemental health]: 11.3% to 4.4%; any in-school services [no in-home or telemental health]: 7.3% to 2.6%).

**Figure.**
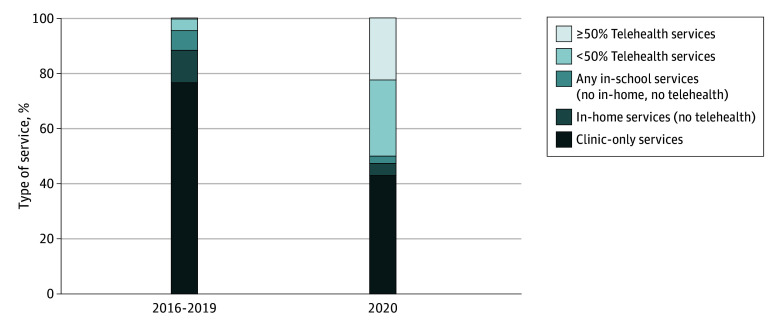
Mental Health Care Setting Among Publicly Insured Children With Depression Data were collected from the T-MSIS analytic files (2016-2020) for 41 states and Washington, District of Columbia. The sample included 799 452 children aged 3 to 17 years with an index depression diagnosis. For children with multiple depression episodes identified, only the first episode was included.

### Receipt of Mental Health Visits

Among children who received services in clinic-only settings, the percentage who received at least 4 mental health visits decreased from 28.3% in 2016 to 2019 to 20.5% in 2020 (*P* < .001) (Table 2). Yet, the percentage who received at least 4 mental health visits increased from 32.7% in 2016 to 2019 to 38.3% in 2020 among children who received some mental health services via telehealth (*P* < .001), and from 7.1% in 2016 to 2019 to 46.3% in 2020 among those who received most services via telemental health (*P* < .001).

**Table 2.** Mental Health Care Use Among Publicly Insured Children With Depression, by Health Care Setting

Outcome	Children, No. (column %)									
	2016-2019					2020				
	Clinic only (n = 538 904)	≤50% Telehealth (n = 29 060)	>50% Telehealth (n = 2829)	Any in-home (n = 79 358)^a^	Any school (n = 51 176)^b^	Clinic only (n = 42 304)	≤50% Telehealth (n = 26 797)	>50% Telehealth (n = 22 115)	Any in-home (n = 4322)^a^	Any school (n = 2587)^b^
Any mental health visit^c^	354 716 (65.8)	24 599 (84.6)	1695 (59.9)	58 887 (74.2)	35 088 (68.6)	24 482 (57.9)^d^	20 657 (77.1)^d^	17 812 (80.5)^d^	3053 (70.6)^d^	1716 (66.3)^d^
≥4 Mental health visits^c^	152 344 (28.3)	9512 (32.7)	201 (7.1)	31 970 (40.3)	17 949 (35.1)	8676 (20.5)^d^	10 255 (38.3)^d^	10 235 (46.3)^d^	1439 (33.3)^d^	751 (29.0)^d^
Any pharmacotherapy^f^	171 641 (31.9)	14 782 (50.9)	1197 (42.3)	19 277 (24.3)	8974 (17.5)	12 638 (29.9)^d^	10 430 (38.9)^d^	6371 (28.8)^d^	908 (21.0)^d^	323 (12.5)^d^
Minimally adequate pharmacotherapy^g^	76 674 (14.2)	6731 (23.2)	615 (21.7)	7916 (10.0)	3512 (6.9)	5912 (14.0)	5492 (20.5)^d^	3193 (14.4)^d^	396 (9.2)	119 (4.6)^d^
Minimally adequate treatment^h^	208 537 (38.7)	13 386 (46.1)	766 (27.1)	36 323 (45.8)	20 098 (39.3)	13 594 (32.1)^d^	13 644 (50.9)^d^	12 043 (54.5)^d^	1685 (39.0)^d^	820 (31.7)^d^

^a^ Any in-home services includes those with at least 1 mental health visit in the home setting, but no telehealth services.

^b^ Any in-school services includes those with at least 1 mental health visit in the school setting, but no telehealth services and no in-home services.

^c^ Measured in the 12 weeks following the index diagnosis.

^d^ *P* < .001 vs 2016 to 2019.

^f^ Measured as any antidepressant medication filled in the 144 days following the index diagnosis.

^g^ Measured as fills or refills of antidepressant medication for 84 of the 144 days following the index diagnosis.

^h^ Measured as the child received at least 4 mental health visits in 12 weeks following index diagnosis and/or minimally adequate pharmacotherapy.

Regression analyses found significant associations between service setting and the receipt of mental health visits (Table 3; eTable 5 in Supplement 1). In 2020, the adjusted percentage of children who received at least 4 mental health visits was 15.3 (95% CI, 13.8-16.8) percentage points higher among those who received some services via telemental health and 22.7 (95% CI, 21.0-24.3) percentage points higher among those who received most services via telemental health, compared with those who received in-clinic only services (model-adjusted predicted probability, 28.1%).

**Table 3.** Adjusted Differences in the Receipt of Mental Health Visits and Pharmacotherapy Among Publicly Insured Children With Depression

Mental health care setting	ME (95% CI), percentage points^a^			
	Any mental health visit^b^	≥4 Mental health visits^b^	Any pharmacotherapy^c^	Minimally adequate pharmacotherapy^d^
**2016-2019**				
Clinic only services, predicted probability, %	66.4	28.1	31.8	14.1
≤50% Telehealth services	13.61 (11.11 to 16.11)	7.60 (5.80 to 9.41)	11.72 (9.54 to 13.90)	5.18 (4.18 to 6.18)
>50% Telehealth services	−7.45 (−10.97 to −3.92)	−18.90 (−20.82 to −16.99)	9.96 (5.39 to 14.54)	7.78 (5.11 to 10.46)
Any in-home services (no telehealth)	7.38 (2.91 to 11.85)	12.66 (8.99 to 16.33)	−5.14 (−6.20 to −4.08)	−2.74 (−3.35 to −2.13)
Any in-school services (no in-home, no telehealth)	1.74 (−2.78 to 6.26)	7.40 (3.93 to 10.87)	−11.75 (−12.98 to −10.52)	−6.32 (−6.93 to −5.71)
**2020**				
Clinic only services, predicted probability, %	59.5	21.2	28.9	13.3
≤50% Telehealth services	14.32 (11.57 to 17.07)	15.28 (13.76 to 16.79)	6.54 (5.70 to 7.37)	4.84 (4.18 to 5.49)
>50% Telehealth services	17.79 (15.37 to 20.22)	22.65 (21.03 to 24.26)	−0.92 (−1.81 to −0.02)	0.69 (0.06 to 1.31)
Any in-home services (no telehealth)	10.16 (6.48 to 13.84)	11.64 (8.85 to 14.43)	−6.18 (−7.94 to −4.43)	−3.37 (−4.57 to −2.17)
Any in-school services (no in-home, no telehealth)	7.77 (0.23 to 15.32)	8.18 (4.52 to 11.84)	−14.36 (−16.36 to −12.37)	−8.08 (−9.12 to −7.03)

Abbreviation: ME, marginal effect.

^a^ Estimates represent the adjusted percentage point change in each outcome variable associated with the setting of interest compared with the reference group, clinic-only care. Regression models controlled for child-level and county-level covariates listed in Table 1 and included state fixed effects, year indicators, and an interaction term between year and health care setting. SEs were clustered at the county level.

^b^ Measured in the 12 weeks (ie, 84 days) following the index diagnosis.

^c^ Measured as any antidepressant medication filled in the 144 days following the index diagnosis.

^d^ Measured as there fills or refills of antidepressant medication for 84 of the 144 days following the index diagnosis.

### Receipt of Minimally Adequate Pharmacotherapy

The percentage who received any pharmacotherapy declined in all settings in 2020 compared with 2016 to 2019 (Table 2). Moreover, the percentage who received minimally adequate pharmacotherapy declined in many settings (Table 2). In 2020, the percentage of children who received minimally adequate pharmacotherapy was 14.0% among those who received clinic-only services, 14.4% among those who received most mental health services via telehealth and 20.5% among those who received some services via telehealth. In regression analyses, the adjusted percentage of children who received minimally adequate pharmacotherapy in 2020 was 4.84 (95% CI, 4.18-5.49) percentage points higher among those who had some visits via telemental health and 0.69 (95% CI, 0.06-1.31) percentage points higher among those who received most visits via telemental health, compared with children who received in-clinic only services (model-adjusted predicted probability, 13.3%). (Table 3; eTable 5 in Supplement 1).

### Receipt of Minimally Adequate Treatment

During the study period, the receipt of minimally adequate depression treatment declined among children who received services in person only (Table 2). Among children receiving clinic-only services, for example, the receipt of any minimally adequate treatment decreased from 38.7% in 2016 to 2019 to 32.1% in 2020 (*P* < .001) (Table 2). In contrast, the percentage of children who received any minimally adequate treatment increased from 2016 to 2019 to 2020 among children receiving some services via telemental health (46.1% to 50.9%; *P* < .001) or most services via telemental health (27.1% to 54.5%; *P* < .001). The increase in minimally adequate treatment among children receiving telemental health care, combined with the steep increase in telemental health services, translated into an overall small increase in the percentage of children who received minimally adequate treatment in the entire sample—from 39.8% during 2016 to 2019 to 42.6% in 2020 (*P* < .001).

In regression analyses, the adjusted percentage of children who received any minimally adequate care in 2020 was 32.3% among those who received services in-clinic only (Table 4; eTable 6 in Supplement 1). This number was 16.1 (95% CI, 14.6-17.6) percentage points higher among those who received some services via telemental health and 19.8 (95% CI, 18.2-21.5) percentage points higher among those who received most services via telemental health.

**Table 4.** Adjusted Differences in Minimally Adequate Depression Treatment Among Publicly Insured Children^a^

Mental health service setting	ME (95% CI), percentage points^b^
**Pre–COVID-19 (2016-2019)**	
Clinic only, predicted probability, %	38.4
≤50% Telehealth	8.62 (6.93 to 10.31)
>50% Telehealth	−6.87 (−9.16 to −4.58)
Any in-home (no telehealth)	9.15 (5.58 to 12.72)
Any in-school (no in-home, no telehealth)	2.36 (−0.90 to 5.62)
**During COVID-19 (2020)**	
Clinic only, predicted probability, %	32.3
≤50% Telehealth	16.07 (14.56 to 17.59)
>50% Telehealth	19.84 (18.20 to 21.47)
Any in-home (no telehealth)	6.92 (4.24 to 9.60)
Any in-school (no in-home, no telehealth)	0.82 (−2.68 to 4.32)

Abbreviation: ME, marginal effect.

^a^ The receipt of minimally adequate depression treatment is a dichotomous indicator for whether the child received at least 4 mental health visits in 12 weeks following index diagnosis or minimally adequate pharmacotherapy.

^b^ Estimates represent the adjusted percentage point change in each outcome variable associated with the setting of interest compared with the reference group, clinic-only care. Regression models controlled for child-level and county-level covariates listed in Table 1 and included state fixed effects, year indicators, and an interaction term between year and health care setting. SEs were clustered at the county level.

### Sensitivity Analyses

Results of analyses that adjusted the *P* values for multiple tests of 5 outcomes using the conservative Bonferroni adjustment (*P* < .01) were generally consistent with 2 exceptions. The findings for children who received most mental health visits via telehealth vs clinic-only settings were significant at the *P* < .05 level when examining the outcome measures of any pharmacotherapy and minimally adequate pharmacotherapy in 2020. Findings from sensitivity analyses conducted among all states and Washinton, DC, regardless of data quality were consistent with the main findings with respect to direction, magnitude, and significance (eTable 7 and eTable 8 in Supplement 1).

## Discussion

To our knowledge, this cross-sectional study is the first study examining whether changes in service delivery settings after the onset of COVID-19 were associated with the receipt of minimally adequate depression treatment among publicly insured children. First, among children with an index depression diagnosis, there were large shifts toward telemental health services in 2020. In addition, those who received some or most mental health services via telehealth in 2020 (vs those who received in-clinic only services) were more likely to receive minimally adequate mental health treatment. Finally, the increase in percentage of children who received telemental health care, combined with the large increases in minimally adequate treatment among those who received any telemental health care, was associated with a small increase in the receipt of any minimally adequate treatment across the entire sample, from 39.8% in 2016 to 2019 to 42.6% in 2020.

Our findings add to the literature on the health care system disruptions and transitions in 2020, including temporary closures of outpatient clinics at the onset of pandemic, as well as the shift to telemental health services during 2020 for children with mental health disorders.^23,49,50^ These results indicate that the disruptions in care had implications for the number of children with an index depression diagnosis in 2020, as this was lower than the mean in the years preceding the pandemic. With respect to health care setting transitions, more than half of children with an index diagnosis in 2020 received any telemental health visit. Furthermore, we documented a decline in other settings in which care was received in person, including clinics, homes, and schools.

Not only was there a significant shift to telemental health care, but the receipt of telemental health services was strongly associated with the receipt of minimally adequate treatment in 2020. For those who received care in person only (including in clinics, homes, and/or schools), the percentage of children who received minimally adequate treatment declined in 2020 compared with the pre–COVID-19 period. One explanation is that there were more disruptions in care for children who relied exclusively on in-person visits, due to safety measures and procedures implemented by health care organizations early in the pandemic.^51^ For example, the National Council on Behavioral Health indicated that more than half of mental health care clinics ceased their programs and almost two-thirds either canceled, rescheduled, or refused patients.^49^ On the patient side, fear of seeking in-person services during the early part of the pandemic may have led to disruptions in mental health visits and/or medication management visits.^52^ On the other hand, the positive association between the telemental health service use and the receipt of any minimally adequate care was partially driven by a large increase in the likelihood of receiving of at least 4 mental health visits. The greater likelihood of receiving at least 4 mental health visits can reflect an advantage telehealth provides with respect to addressing the logistics of psychotherapy and psychosocial services, including frequency of sessions, distance to mental health practitioner, transportation availability, and family schedules.^19,20,39,53^

The substantially higher likelihood of receiving minimally adequate treatment among children who had some or most visits via telemental health translated into a small but significant increase in the likelihood of receiving any minimally adequate care in 2020 in the entire sample compared with earlier years. It is notable that there was any increase in the percentage of children receiving minimally adequate care during a time when there were significant disruptions in care.^49,50^ These findings highlight the potential of hybrid depression care, in which at least some services are delivered via telemental health, to help publicly insured children receive enough services, such that there is an opportunity to gain clinical benefit. Prior to COVID-19, very few states allowed Medicaid beneficiaries to receive telehealth services in their homes.^24^ During 2020, flexibilities to telehealth reimbursement policies were instituted in Medicaid programs nationwide.^24^ Yet, after the COVID-19 public health emergency ended in May 2023, flexibilities in these policies began to sunset in some states^24^ and gave rise to increased state variability in continued coverage for various telehealth modalities.^54,55^ Future research will be needed to monitor the receipt of hybrid care among publicly insured children in need of depression treatment and whether hybrid care continues to facilitate higher rates of minimally adequate treatment.

### Limitations

There are several study limitations. First, causality in the associations of interest cannot be established due to the observational study design. Second, although the analyses included measures of comorbid mental health disorders, there remain unmeasured differences in the presentation and severity of depression in the sample. Prior studies have reported that individuals with more severe mental health symptoms are less likely to receive care via telemental health,^56,57^ while mental health symptom severity is positively associated with the receipt of minimally adequate care.^18^ To the extent that this is true in our sample, the associations that were estimated between telemental health service utilization and the receipt of minimally adequate care would be biased toward the null hypothesis; in other words, stronger measurement of clinical severity may yield larger effect sizes for these associations. Third, we only included the first depression episode among children in our sample; thus findings may not generalize to additional episodes. Fourth, administrative claims data can include measurement errors because the data are generated for billing purposes.^58,59,60,61^ Moreover, there is variation in the quality of the T-MSIS analytic files data that were used for this study.^33,62^ To address this limitation, our primary analysis was conducted with 41 states and Washington, DC, that do not have high concerns in the other service files and the pharmacy files.

## Conclusions

The findings of this cross-sectional study highlight the extent to which publicly insured children with a depression diagnosis received telemental health services in 2020 compared with earlier years. These findings also demonstrate large and significant associations between receipt of telemental health services and the receipt of minimally adequate depression treatment after the onset of the COVID-19 pandemic. Continued coverage and monitoring of telemental health use by state Medicaid and CHIP programs is critical to facilitate access and minimize barriers to mental health services.

## Notes

Supplement 1. **eTable 1.** Diagnosis Codes for Mental Health Conditions **eTable 2.** Procedure Codes for Mental Health Visits **eTable 3.** Antidepressants Used to Identify Medication Fills and Refills **eTable 4.** Sample Characteristics among Publicly Insured Children Diagnosed with Depression, by Health Care Settings **eTable 5.** Adjusted Differences in the Receipt of Mental Health Visits and Pharmacotherapy Among Publicly Insured Children With Depression **eTable 6.** Adjusted Differences in Minimally Adequate Depression Treatment Among Publicly Insured Children **eTable 7.** Sensitivity Analysis: Adjusted Differences in the Receipt of Mental Health Visits and Pharmacotherapy Among Publicly Insured Children With Depression in 50 States and Washington D.C. **eTable 8.** Sensitivity Analysis: Adjusted Differences in Minimally Adequate Depression Treatment Among Publicly Insured Children in 50 States and Washington D.C. **eFigure.** Sample Derivation Process

Supplement 2.
Data Sharing Statement

## References

[1] Centers for Disease Control and Prevention. Youth Risk Behavior Survey data summary & trends report: 2013–2023. Accessed December 20, 2024. https://www.cdc.gov/yrbs/dstr/pdf/YRBS-2023-Data-Summary-Trend-Report.pdf

[2] Agency for Healthcare Research and Quality. 2022 National healthcare quality and disparities report. 2022. Accessed October 2, 2024. https://www.ahrq.gov/research/findings/nhqrdr/nhqdr22/index.html 36475568

[3] Daly M. Prevalence of depression among adolescents in the U.S. from 2009 to 2019: analysis of trends by sex, race/ethnicity, and income. J Adolesc Health. 2022;70(3):496-499. doi:10.1016/j.jadohealth.2021.08.026 34663534

[4] Substance Abuse and Mental Health Services Administration. Key substance use and mental health indicators in the United States: results from the 2022 National Survey on Drug Use and Health. Accessed October 2, 2025. https://www.samhsa.gov/data/report/2022-nsduh-annual-national-report

[5] McLaughlin KA, Greif Green J, Gruber MJ, Sampson NA, Zaslavsky AM, Kessler RC. Childhood adversities and first onset of psychiatric disorders in a national sample of US adolescents. Arch Gen Psychiatry. 2012;69(11):1151-1160. doi:10.1001/archgenpsychiatry.2011.2277 23117636 PMC3490224

[6] Lee D, McLanahan S. Family structure transitions and child development: instability, selection, and population heterogeneity. Am Sociol Rev. 2015;80(4):738-763. doi:10.1177/0003122415592129 27293242 PMC4902167

[7] Quan X, Lei H, Zhu C, Wang Y, Lu F, Zhang C. Family income and child depression: the chain mediating effect of parental involvement, children’s self-esteem, and group differences. Children (Basel). 2024;11(4):478. doi:10.3390/children11040478 38671695 PMC11048797

[8] Cornachione E, Rudowitz R, Artiga S. Children’s health coverage: the role of Medicaid and CHIP and issues for the future. *Kaiser Family Foundation*. June 27, 2016. Accessed October 4, 2024. https://www.kff.org/report-section/childrens-health-coverage-the-role-of-medicaid-and-chip-and-issues-for-the-future-issue-brief/

[9] Medicaid enrollment and unwinding tracker. *Kaiser Family Foundation*. May 2, 2025. Accessed May 8, 2025. https://www.kff.org/report-section/medicaid-enrollment-and-unwinding-tracker-enrollment-data/

[10] Walter HJ, Abright AR, Bukstein OG, . Clinical practice guideline for the assessment and treatment of children and adolescents with major and persistent depressive disorders. J Am Acad Child Adolesc Psychiatry. 2023;62(5):479-502. doi:10.1016/j.jaac.2022.10.001 36273673

[11] Birmaher B, Brent D, Bernet W, ; AACAP Work Group on Quality Issues. Practice parameter for the assessment and treatment of children and adolescents with depressive disorders. J Am Acad Child Adolesc Psychiatry. 2007;46(11):1503-1526. doi:10.1097/chi.0b013e318145ae1c 18049300

[12] American Psychological Association. Clinical practice guideline for the treatment of depression across three age cohorts. Accessed May 3, 2025. https://www.apa.org/depression-guideline

[13] Cummings JR, Ji X, Lally C, Druss BG. Racial and ethnic differences in minimally adequate depression care among Medicaid-enrolled youth. J Am Acad Child Adolesc Psychiatry. 2019;58(1):128-138. doi:10.1016/j.jaac.2018.04.025 30577928 PMC8051617

[14] Stein BD, Sorbero MJ, Dalton E, . Predictors of adequate depression treatment among Medicaid-enrolled youth. Soc Psychiatry Psychiatr Epidemiol. 2013;48(5):757-765. doi:10.1007/s00127-012-0593-7 23589098

[15] Davis NO, Jones KA, French A, . Treatment and outcomes among North Carolina Medicaid-insured youth with depression. JAACAP Open. 2023;1(3):196-205. doi:10.1016/j.jaacop.2023.06.002 39552703 PMC11562409

[16] Gulliver A, Griffiths KM, Christensen H. Perceived barriers and facilitators to mental health help-seeking in young people: a systematic review. BMC Psychiatry. 2010;10:113. doi:10.1186/1471-244X-10-113 21192795 PMC3022639

[17] Bishop TF, Press MJ, Keyhani S, Pincus HA. Acceptance of insurance by psychiatrists and the implications for access to mental health care. JAMA Psychiatry. 2014;71(2):176-181. doi:10.1001/jamapsychiatry.2013.2862 24337499 PMC3967759

[18] Cummings JR, Ji X, Druss BG. Mental health service use by Medicaid-enrolled children and adolescents in primary care safety-net clinics. Psychiatr Serv. 2020;71(4):328-336. doi:10.1176/appi.ps.201800540 31960778 PMC8052634

[19] Ofonedu ME, Belcher HME, Budhathoki C, Gross DA. Understanding barriers to initial treatment engagement among underserved families seeking mental health services. J Child Fam Stud. 2017;26(3):863-876. doi:10.1007/s10826-016-0603-6 28584498 PMC5456294

[20] Owens PL, Hoagwood K, Horwitz SM, . Barriers to children’s mental health services. J Am Acad Child Adolesc Psychiatry. 2002;41(6):731-738. doi:10.1097/00004583-200206000-00013 12049448

[21] Cummings JR, Song M, Gaydos LM, Blake SC. Stakeholder perspectives on the advantages and challenges of expanded school mental health services for publically-insured youth. Psychol Serv. 2023;20(3):647-656. doi:10.1037/ser0000590 34793190

[22] Centers for Disease Control and Prevention. Improving access to children’s mental health care. Accessed October 2, 2024. https://archive.cdc.gov/www_cdc_gov/childrensmentalhealth/access.html

[23] Ali MM, West KD, Bagalman E, Sherry TB. Telepsychiatry use before and during the COVID-19 pandemic among children enrolled in Medicaid. Psychiatr Serv. 2023;74(6):644-647. doi:10.1176/appi.ps.20220378 36444530

[24] Rudich J, Conmy AB, Chu RC, Peters C, De Lew N, Sommers BD. State Medicaid telehealth policies before and during the COVID-19 public health emergency: 2022 updates (Issue Brief No. HP2022-29). Accessed October 2, 2024. https://aspe.hhs.gov/sites/default/files/documents/190b4b132f984db14924cbad00d19cce/Medicaid-Telehealth-IB-Update-Final.pdf

[25] Wilk AS, Hu JC, Wen H, Cummings JR. Recent trends in school-based mental health services among low-income and racial and ethnic minority adolescents. JAMA Pediatr. 2022;176(8):813-815. doi:10.1001/jamapediatrics.2022.1020 35499844 PMC9062765

[26] Richards MC, Benson NM, Kozloff N, Franklin MS. Remodeling broken systems: addressing the national emergency in child and adolescent mental health. Psychiatr Serv. 2024;75(3):291-293. doi:10.1176/appi.ps.20220283 37711021

[27] Cummings JR, Shellman MH, Stein BD, Asplund J, Lin H, Serban N. Association between in-home treatment and engagement in psychosocial services among Medicaid-enrolled youth. J Am Acad Child Adolesc Psychiatry. 2022;61(11):1351-1361. doi:10.1016/j.jaac.2022.03.028 35427731

[28] Centers for Medicare & Medicaid Services. Medicaid Analytic eXtract (MAX) general information. Accessed November 10, 2024. https://www.cms.gov/data-research/computer-data-systems/medicaid-data-sources-general-information/medicaid-analytic-extract-max-general-information

[29] Chronic Condition Data Warehouse. CCW user guide: T-MSIS analytic files (TAF) research identifiable files (RIFs). Updated November 2019. Accessed February 17, 2020. https://www2.ccwdata.org/documents/10280/19002246/ccw-taf-rif-user-guide.pdf

[30] Health Resources and Services Administration, U.S. Department of Health and Human Services. Area health resources file. Accessed December 20, 2024. https://data.hrsa.gov/topics/health-workforce/ahrf

[31] Robert Graham Center - Policy Studies in Family Medicine & Primary Care. Social Deprivation Index (SDI). Updated November 5, 2018. Accessed January 13, 2025. https://www.graham-center.org/maps-data-tools/social-deprivation-index.html

[32] Federal Communications Commission. FCC form 477 additional data. Accessed January 13, 2025. https://www.fcc.gov/general/fcc-form-477-additional-data

[33] Medicaid.gov. DQATLAS. Accessed December 20, 2024. https://www.medicaid.gov/dq-atlas/welcome

[34] McDanal R, Parisi D, Opara I, Schleider JL. Effects of brief interventions on internalizing symptoms and substance use in youth: a systematic review. Clin Child Fam Psychol Rev. 2022;25(2):339-355. doi:10.1007/s10567-021-00372-2 34731373 PMC9061892

[35] Mehrotra A, Huskamp HA, Souza J, . Rapid growth in mental health telemedicine use among rural Medicare beneficiaries, wide variation across states. Health Aff (Millwood). 2017;36(5):909-917. doi:10.1377/hlthaff.2016.1461 28461359

[36] Zhao X, Bhattacharjee S, Innes KE, LeMasters TJ, Dwibedi N, Sambamoorthi U. The impact of telemental health use on healthcare costs among commercially insured adults with mental health conditions. Curr Med Res Opin. 2020;36(9):1541-1548. doi:10.1080/03007995.2020.1790345 32609549 PMC7535072

[37] Chronic Conditions Warehouse Virtual Data Center. Data dictionaries. Accessed November 14, 2024. https://www2.ccwdata.org/web/guest/data-dictionaries

[38] Hu X, Graetz I, Marchak JG, Mertens AC, Ji X, Cummings JR. Racial and ethnic disparities in telemental health use among publicly insured children. Am J Manag Care. 2025;31(3):119-126. doi:10.37765/ajmc.2025.89674 40053404

[39] Cummings JR, Kalk T, Trello S, Walker ER, Graetz I. Therapists’ perspectives on access to telemental health among Medicaid-enrolled youth. Am J Manag Care. 2023;29(11):e339-e347. doi:10.37765/ajmc.2023.89430 37948654

[40] Andersen RM, Davidson PL. Improving access to care in America: individual and contextual indicators. In: Andersen RM, Rice TH, Kominski GF, . Changing the U.S. Health Care System: Key Issues in Health Services Policy and Management. 3rd ed. Jossey-Bass; 2007:3-31.

[41] Saunders H, Chidambaram P. Medicaid administrative data: challenges with race, ethnicity, and other demographic variables. Kaiser Family Foundation. Apr 28, 2022. Accessed November 14, 2024. https://www.kff.org/medicaid/issue-brief/medicaid-administrative-data-challenges-with-race-ethnicity-and-other-demographic-variables/

[42] Reed ME, Huang J, Graetz I, . Patient characteristics associated with choosing a telemedicine visit vs office visit with the same primary care clinicians. JAMA Netw Open. 2020;3(6):e205873. doi:10.1001/jamanetworkopen.2020.5873 32585018 PMC7301227

[43] McClellan M, Rajkumar R, Couch M, . Health care payers COVID-19 impact assessment: lessons learned and compelling needs. NAM Perspect. Published online May 17, 2021. doi:10.31478/202105a 34532685 PMC8406497

[44] Shaver J. The state of telehealth before and after the COVID-19 pandemic. Prim Care. 2022;49(4):517-530. doi:10.1016/j.pop.2022.04.002 36357058 PMC9035352

[45] Weigel G, Ramaswamy A, Sobel L, Salganicoff A, Cubanski J. Opportunities and barriers for telemedicine in the U.S. during the COVID-19 emergency and beyond. *Kaiser Family Foundation*. May 11, 2020. Accessed January 13, 2025. https://www.kff.org/womens-health-policy/issue-brief/opportunities-and-barriers-for-telemedicine-in-the-u-s-during-the-covid-19-emergency-and-beyond/

[46] Norton EC, Dowd BE, Maciejewski ML. Marginal effects-quantifying the effect of changes in risk factors in logistic regression models. JAMA. 2019;321(13):1304-1305. doi:10.1001/jama.2019.1954 30848814

[47] SAS Institute. Predictive margins and average marginal effects. Accessed December 20, 2024. http://support.sas.com/kb/63/038.html

[48] ResDAC. CCW Virtual Research Data Center (VRDC). Accessed December 20, 2024. https://resdac.org/cms-virtual-research-data-center-vrdc

[49] National Council for Behavioral Health. National Council for Behavioral Health member survey: impact of COVID-19 on behavioral health organizations. Published online September 2020. Accessed December 20, 2024. https://www.thenationalcouncil.org/wp-content/uploads/2022/11/NCBH_Member_Survey_Sept_2020_CTD2.pdf

[50] Panchal N, Kamal R, Cox C, Garfield R, Chidambaram P. Mental health and substance use considerations among children during the COVID-19 pandemic. *Kaiser Family Foundation*. May 26, 2021. Accessed May 6, 2025. https://www.kff.org/mental-health/issue-brief/mental-health-and-substance-use-considerations-among-children-during-the-covid-19-pandemic/

[51] Stocking JC, Sandrock C, Fitall E, Hall KK, Gale B. AHRQ PSNet annual perspective: impact of the COVID-19 pandemic on patient safety. Published online August 31, 2020. Accessed January 13, 2025. https://psnet.ahrq.gov/perspective/ahrq-psnet-annual-perspective-impact-covid-19-pandemic-patient-safety

[52] Anderson KE, McGinty EE, Presskreischer R, Barry CL. Reports of forgone medical care among US adults during the initial phase of the COVID-19 pandemic. JAMA Netw Open. 2021;4(1):e2034882. doi:10.1001/jamanetworkopen.2020.34882 33475757 PMC7821029

[53] Radez J, Reardon T, Creswell C, Lawrence PJ, Evdoka-Burton G, Waite P. Why do children and adolescents (not) seek and access professional help for their mental health problems: a systematic review of quantitative and qualitative studies. Eur Child Adolesc Psychiatry. 2021;30(2):183-211. doi:10.1007/s00787-019-01469-4 31965309 PMC7932953

[54] Cubanski J, Kates J, Tolbert J, Guth M, Pollitz K. What happens when COVID-19 emergency declarations end: implications for coverage, costs, and access. *Kaiser Family Foundation*. January 31, 2023. Accessed January 13, 2025. https://www.kff.org/coronavirus-covid-19/issue-brief/what-happens-when-covid-19-emergency-declarations-end-implications-for-coverage-costs-and-access/

[55] Hinton E, Guth M, Raphael J, . How the pandemic continues to shape Medicaid priorities: results from an annual Medicaid budget survey for state fiscal years 2022 and 2023—telehealth—10030. *Kaiser Family Foundation*. October 25, 2022. Accessed January 13, 2025. https://www.kff.org/report-section/medicaid-budget-survey-for-state-fiscal-years-2022-and-2023-telehealth/

[56] Hutchison M, Russell BS, Gans KM, Starkweather AR. Online administration of a pilot mindfulness-based intervention for adolescents: feasibility, treatment perception and satisfaction. Curr Psychol. 2022;42(22):1-13. doi:10.1007/s12144-022-03025-x 35382039 PMC8972985

[57] Neumann A, König HH, Bokermann J, Hajek A. Determinants of patient use and satisfaction with synchronous telemental health services during the COVID-19 pandemic: systematic review. JMIR Ment Health. 2023;10:e46148. doi:10.2196/46148 37594785 PMC10474517

[58] Funk MJ, Landi SN. Misclassification in administrative claims data: quantifying the impact on treatment effect estimates. Curr Epidemiol Rep. 2014;1(4):175-185. doi:10.1007/s40471-014-0027-z 26085977 PMC4465810

[59] Khwaja HA, Syed H, Cranston DW. Coding errors: a comparative analysis of hospital and prospectively collected departmental data. BJU Int. 2002;89(3):178-180. doi:10.1046/j.1464-4096.2001.01428.x 11856094

[60] Woodworth GF, Baird CJ, Garces-Ambrossi G, Tonascia J, Tamargo RJ. Inaccuracy of the administrative database: comparative analysis of two databases for the diagnosis and treatment of intracranial aneurysms. Neurosurgery. 2009;65(2):251-256. doi:10.1227/01.NEU.0000347003.35690.7A 19625902

[61] Johnson EK, Nelson CP. Values and pitfalls of the use of administrative databases for outcomes assessment. J Urol. 2013;190(1):17-18. doi:10.1016/j.juro.2013.04.048 23608038 PMC4114235

[62] Gordon S, Johnson A, Kennedy S, McConnell J, Schpero W. Catalyzing Medicaid Policy Research with T-MSIS Analytic Files (TAF): Learnings From Year 1 of the Medicaid Data Learning Network (MDLN). AcademyHealth; 2023.

