# Supplementary material for: Health Care Setting and Minimally Adequate Depression Treatment Among Publicly Insured Children
Source: JAMA Netw Open. 2025 Aug 28;8(8):e2528345. doi: 10.1001/jamanetworkopen.2025.28345 (PMC12395313; doi:10.1001/jamanetworkopen.2025.28345)
Supplement: Supplement 1. — eTable 1. Diagnosis Codes for Mental Health Conditions eTable 2. Procedure Codes for Mental Health Visits eTable 3. Antidepressants Used to Identify Medication Fills and Refills eTable 4. Sample Characteristics among Publicly Insured Children Diagnosed with Depression, by Health Care Settings eTable 5. Adjusted Differences in the Receipt of Mental Health Visits and Pharmacotherapy Among Publicly Insured Children With Depression eTable 6. Adjusted Differences in Minimally Adequate Depression Treatment Among Publicly Insured Children eTable 7. Sensitivity Analysis: Adjusted Differences in the Receipt of Mental Health Visits and Pharmacotherapy Among Publicly Insured Children With Depression in 50 States and Washington D.C. eTable 8. Sensitivity Analysis: Adjusted Differences in Minimally Adequate Depression Treatment Among Publicly Insured Children in 50 States and Washington D.C. eFigure. Sample Derivation Process [file jamanetwopen-e2528345-s001.pdf]

Supplemental Online Content

Cummings J, Hu X, Graetz I, Marchak J, Ramos C, Ji X. Health care setting and minimally adequate depression treatment among publicly insured children. *JAMA Netw Open*. 2025;8(8):e2528345. doi:10.1001/jamanetworkopen.2025.28345

eTable 1. Diagnosis Codes for Mental Health Conditions

eTable 2. Procedure Codes for Mental Health Visits

eTable 3. Antidepressants Used to Identify Medication Fills and Refills

eTable 4. Sample Characteristics among Publicly Insured Children Diagnosed with Depression, by Health Care Settings

eTable 5. Adjusted Differences in the Receipt of Mental Health Visits and Pharmacotherapy Among Publicly Insured Children With Depression

eTable 6. Adjusted Differences in Minimally Adequate Depression Treatment Among Publicly Insured Children

eTable 7. Sensitivity Analysis: Adjusted Differences in the Receipt of Mental Health Visits and Pharmacotherapy Among Publicly Insured Children With Depression in 50 States and Washington D.C.

eTable 8. Sensitivity Analysis: Adjusted Differences in Minimally Adequate Depression Treatment Among Publicly Insured Children in 50 States and Washington D.C.

eFigure. Sample Derivation Process

This supplemental material has been provided by the authors to give readers additional information about their work.

© 2025 Cummings J et al. *JAMA Network Open*.

eTable 1. ICD -10 Diagnosis Codes for Mental Health Conditions

Mental Health Conditions	Diagnosis Codes
ADHD	F90.0, F90.1, F90.2, F90.8, F90.9
Anxiety	F40.00, F40.01, F40.02, F40.10, F40.11, F40.210, F40.218, F40.220, F40.228, F40.230, F40.231, F40.232, F40.233, F40.240, F40.241, F40.242, F40.243, F40.248, F40.290, F40.291, F40.298, F40.8, F40.9, F41.0, F41.1, F41.3, F41.8, F41.9, F06.4, F93.0, F94.0
Autism	F84.5, F84.0
Depressive disorder	F06.31, F06.32, F32.0, F32.1, F32.2, F32.3, F32.4, F32.5, F32.81, F32.9, F33.0, F33.1, F33.2, F33.3, F33.40, F33.41, F33.42, F33.9, F34.1, F34.81, F32.89, F33.8, F32.8
Disruptive disorder	F60.2, F63.1, F63.2, F63.81, F63.89, F63.9, F91.0, F91.1, F91.2, F91.8, F91.9, F91.3
Obsessive-compulsive and related	F63.3, F42, F42.2, F42.3, F42.8, F42.9, F42.4
Trauma & stressor	F43.0, F43.10, F43.11, F43.12, F43.20, F43.21, F43.22, F43.23, F43.24, F43.25, F43.29, F43.8, F43.9, F94.1, F94.2, R45.7
Other mental health disorder	F50.00, F50.01, F50.02, F50.2, F50.8, F50.81, F50.82, F50.89, F50.9, F98.21, F98.29, F98.3, F06.0, F06.2, F20.0, F20.1, F20.2, F20.3, F20.5, F20.81, F20.89, F20.9, F21, F22, F23, F24, F25.0, F25.1, F25.8, F25.9, F28, F29, F60.1, F06.33, F06.34, F30.10, F30.11, F30.12, F30.13, F30.2, F30.3, F30.4, F30.8, F30.9, F31.0, F31.10, F31.11, F31.12, F31.13, F31.2, F31.30, F31.31, F31.32, F31.4, F31.5, F31.60, F31.61, F31.62, F31.63, F31.64, F31.70, F31.71, F31.72, F31.73, F31.74, F31.75, F31.76, F31.77, F31.78, F31.81, F31.89, F31.9, F34.0, F60.0, F60.4, F60.5, F60.6, F60.7, F60.81, F60.89, F60.9, F69, F60.3, F70, F71, F72, F73, F78, F79, F80.0, F80.1, F80.2, F80.4, F80.81, F80.82, F80.89, F80.9, F81.0, F81.2, F81.81, F81.89, F81.9, F82, F84.3, F84.8, F84.9, F84.2, F88, F89, F95.0, F95.1, F95.2, F95.8, F95.9, F98.4, F98.5, R48.0, R41.83, F06.30, F34.8, F34.89, F34.9, F39, F48.8, F48.9, F64.2, F93.8, F93.9, F94.8, F94.9, F98.0, F98.1, F98.8, F98.9

© 2025 Cummings J et al. *JAMA Network Open*.

eTable 2. Procedure Codes for Mental Health Visits

Category of Psychosocial Services	Procedure Codes
Applied behavior analysis	0359T, 0360T, 0361T, 0362T, 0363T, 0364T, 0365T, 0366T, 0367T, 0368T, 0369T, 0370T, 0371T, 0372T, 0373T, 0374T, 97153, 97154, 97155, 97156, 97157, 97158
Psychosocial intervention	90804, 90805, 90806, 90807, 90808, 90809, 90810, 90811, 90812, 90813, 90814, 90815, 90833, 90838, 90862, 90863, 90832, 90834, 90836, 90837, 90839, 90840, 90845, 90846, 90847, 90849, 90853, 90857, 90875, 90876, 96153, 96164, 96165, 96152, 96154, 96155, 96158, 96159, 96167, 96168, 96170, 96171, 99354, 99355, 99510, G0410, G0411, H0004, H2012, H2033
Medication management	90792, M0064
Mental health assessment	90791, 90801, 90802, 96127, 96150, 96151, 96156, 96160, 96161, 97151, 97152, G0444, H0031
Neuropsychological & psychological testing	96100, 96101, 96102, 96103, 96116, 96117, 96118, 96119, 96120, 96121, 96125, 96130, 96131, 96132, 96133, 96136, 96137, 96138, 96139, 96146
Other mental health intervention	90880, 97533, G0409, G0469, G0470, G0515, H0036, H0037, H0046, H2014, H2015, H2016, H2017, H2018, H2019, H2020, H2021, H2022, H2027, H2030, H2031, H2032, S9480, S9484, S9485, T1027

© 2025 Cummings J et al. *JAMA Network Open*.

eTable 3. Antidepressants Used to Identify Medication Fills and Refills

Generic Drug Name	
Amantadine HCl;Amitriptyline HCl;Cyclobenzaprine H	Levomilnacipran Hydrochloride
Amitriptyline HCl;Cream, Multi Ingredient	Levomilnacipran Hydrochloride;Levomilnacipran Hydr
Amitriptyline HCl;Medical Food	Maprotiline Hydrochloride
Amitriptyline Hydrochloride	Mirtazapine
Amitriptyline Hydrochloride/Chlordiazepoxide	Nefazodone Hydrochloride
Amitriptyline Hydrochloride/Perphenazine	Nortriptyline Hydrochloride
Amoxapine	Paroxetine Hydrochloride
Bupropion Hydrochloride	Paroxetine Mesylate
Chlorprothixene	Phenelzine Sulfate
Citalopram Hydrobromide	Protriptyline Hydrochloride
Clomipramine Hydrochloride	Selegiline
Desipramine Hydrochloride	Selegiline Hydrochloride
Desvenlafaxine	Sertraline Hydrochloride
Desvenlafaxine Succinate	Tranylcypromine Sulfate
Doxepin Hydrochloride	Trazodone Hydrochloride
Duloxetine Hydrochloride	Trimipramine Maleate
Duloxetine Hydrochloride;Lidocaine/Menthol	Venlafaxine Hydrochloride
Escitalopram Oxalate	Vilazodone HCl;Vilazodone HCl;Vilazodone HCl
Fluoxetine HCl;Medical Food	Vilazodone Hydrochloride
Fluoxetine Hydrochloride	Vilazodone Hydrochloride;Vilazodone Hydrochloride
Fluvoxamine Maleate	Vortioxetine Hydrobromide
Imipramine Hydrochloride	buPROPion hydrobromide
Imipramine Pamoate	buPROPion hydrochloride;Medical Food
Inactive Record	traZODone hydrochloride;Medical Food
Isocarboxazid	

© 2025 Cummings J et al. *JAMA Network Open*.

eTable 4. Sample Characteristics of Publicly Insured Children with Index Depression Diagnosis, by Health Care Setting

	2016-2019										2020									
	Clinic Only n=538,904		Some Telehealth n=29,060		Majority Telehealth n=2,829		Any In-Home[1] n=79,358		Any School[2] n=51,176		Clinic Only n=42,304		Some Telehealth n=26,797		Majority Telehealth n=22,115		Any In-Home[1] n=4,322		Any School[2] n=2,587	
	n	%	n	%	n	%	n	%	n	%	n	%	n	%	n	%	n	%	n	%
Individual-Level Covariates																				
Age group*																				
3-11 years	99264	18.4	5511	19.0	715	25.3	21686	27.3	14261	27.9	6491	15.3	4954	18.5	4223	19.1	998	23.1	611	23.6
12-17 years	439640	81.6	23549	81.0	2114	74.7	57672	72.7	36915	72.1	35813	84.7	21843	81.5	17892	80.9	3324	76.9	1976	76.4
Female Sex*	321196	59.6	16405	56.5	1363	48.2	42995	54.2	26982	52.7	26265	62.1	16237	60.6	13762	62.2	2425	56.1	1384	53.5
Race/Ethnicity[1,*]																				
Hispanic	144421	26.8	7306	25.1	609	21.5	22662	28.6	16748	32.7	10447	24.7	6862	25.6	5982	27.0	1114	25.8	853	33.0
Non-Hispanic Amer.Indian/Alaskan Native	11713	2.2	750	2.6	74	2.6	1104	1.4	1092	2.1	928	2.2	442	1.6	297	1.3	78	1.8	37	1.4
Non-Hispanic Asian/Pacific Islander	10610	2.0	244	0.8	33	1.2	1176	1.5	1039	2.0	748	1.8	409	1.5	372	1.7	89	2.1	35	1.4
Non-Hispanic Black	74900	13.9	3364	11.6	384	13.6	17802	22.4	5424	10.6	5848	13.8	3516	13.1	3170	14.3	1010	23.4	375	14.5
Non-Hispanic multi-racial group/Unknown	94641	17.6	6227	21.4	701	24.8	14581	18.4	9397	18.4	6916	16.3	4913	18.3	3973	18.0	466	10.8	436	16.9
Non-Hispanic White	202619	37.6	11169	38.4	1028	36.3	22033	27.8	17476	34.1	17417	41.2	10655	39.8	8321	37.6	1565	36.2	851	32.9
Medicaid Eligibility Type[2,*]																				
Low-income	488625	90.7	25812	88.8	2424	85.7	68669	86.5	45934	89.8	38879	91.9	24915	93.0	20724	93.7	3832	88.7	2376	91.8
Disability	34362	6.4	2735	9.4	366	12.9	8677	10.9	3937	7.7	2281	5.4	1558	5.8	1216	5.5	439	10.2	141	5.5
Other/Unknown	15917	3.0	513	1.8	39	1.4	2012	2.5	1305	2.6	1144	2.7	324	1.2	175	0.8	51	1.2	70	2.7
Plan Type[3,*]																				
Comprehensive managed care organization	436475	81.0	24560	84.5	2433	86.0	61906	78.0	34906	68.2	34995	82.7	21694	81.0	18537	83.8	3625	83.9	2024	78.2
Primary care case management	21183	3.9	1076	3.7	116	4.1	1469	1.9	2706	5.3	1611	3.8	1264	4.7	618	2.8	41	0.9	175	6.8
Prepaid health plan	43537	8.1	2426	8.3	169	6.0	10039	12.7	10409	20.3	3400	8.0	2863	10.7	2386	10.8	435	10.1	283	10.9
Other/Unknown	37709	7.0	998	3.4	111	3.9	5944	7.5	3155	6.2	2298	5.4	976	3.6	574	2.6	221	5.1	105	4.1
Co-existing Conditions																				
Attention deficit hyperactivity disorder (yes/no)*	63568	11.8	6903	23.8	882	31.2	13348	16.8	6767	13.2	4149	9.8	4149	15.5	2956	13.4	610	14.1	228	8.8
Anxiety disorder (yes/no)*	97468	18.1	6780	23.3	471	16.6	9645	12.2	6300	12.3	8716	20.6	6632	24.7	4985	22.5	680	15.7	334	12.9
Autism (yes/no)*	5398	1.0	595	2.0	40	1.4	1857	2.3	676	1.3	477	1.1	576	2.1	263	1.2	130	3.0	27	1.0
Disruptive, impulse control, and conduct disorders (yes/no)*	30881	5.7	3461	11.9	179	6.3	8560	10.8	3343	6.5	2071	4.9	2047	7.6	1036	4.7	405	9.4	83	3.2
Trauma and other stressor-related disorders (yes/no)*	54654	10.1	5748	19.8	290	10.3	11068	13.9	4370	8.5	4254	10.1	4434	16.5	2682	12.1	585	13.5	215	8.3
Other mental health conditions (yes/no)*	45690	8.5	5217	18.0	195	6.9	10477	13.2	6212	12.1	2662	6.3	2992	11.2	1230	5.6	394	9.1	274	10.6

© 2025 Cummings J et al. *JAMA Network Open*.

eTable 4 (Cont.). Sample Characteristics of Publicly Insured Children with Index Depression Diagnosis, by Health Care Setting

	2016-2019										2020									
	Clinic Only n=538,904		Some Telehealth n=29,060		Majority Telehealth n=2,829		Any In-Home n=79,358		Any School n=51,176		Clinic Only n=42,304		Some Telehealth n=26,797		Majority Telehealth n=22,115		Any In-Home n=4,322		Any School n=2,587	
	n	%	n	%	n	%	n	%	n	%	n	%	n	%	n	%	n	%	n	%
County-Level Covariates																				
Percent Non-Hispanic Black Population[4,*] Quartiles																				
Q1	40651	7.5	3897	13.4	285	10.1	4221	5.3	4035	7.9	3105	7.3	2045	7.6	1618	7.3	272	6.3	156	6.0
Q2	93341	17.3	4744	16.3	405	14.3	7699	9.7	9137	17.9	7484	17.7	4628	17.3	3352	15.2	665	15.4	477	18.4
Q3	199630	37.0	12313	42.4	1258	44.5	30743	38.7	25046	48.9	15711	37.1	11126	41.5	8966	40.5	1883	43.6	1241	48.0
Q4	205282	38.1	8106	27.9	881	31.1	36695	46.2	12958	25.3	16004	37.8	8998	33.6	8179	37.0	1502	34.8	713	27.6
Percent Hispanic Population[4,*], Quartiles																				
Q1	50933	9.5	2523	8.7	201	7.1	6545	8.2	3355	6.6	3749	8.9	2014	7.5	1334	6.0	285	6.6	168	6.5
Q2	65609	12.2	3521	12.1	327	11.6	9695	12.2	7153	14.0	5494	13.0	3484	13.0	2497	11.3	451	10.4	396	15.3
Q3	126022	23.4	4995	17.2	414	14.6	19535	24.6	11853	23.2	9799	23.2	6312	23.6	5040	22.8	1174	27.2	556	21.5
Q4	296340	55.0	18021	62.0	1887	66.7	43583	54.9	28815	56.3	23262	55.0	14987	55.9	13244	59.9	2412	55.8	1467	56.7
Social Deprivation Index[5,*], Quartiles																				
Q1	69995	13.0	2247	7.7	230	8.1	8121	10.2	4719	9.2	5533	13.1	3138	11.7	2658	12.0	594	13.7	228	8.8
Q2	113637	21.1	4391	15.1	430	15.2	11615	14.6	11697	22.9	8600	20.3	5257	19.6	4213	19.1	912	21.1	534	20.6
Q3	145130	26.9	8872	30.5	830	29.3	19694	24.8	13410	26.2	10663	25.2	7065	26.4	5592	25.3	1034	23.9	631	24.4
Q4	210142	39.0	13550	46.6	1339	47.3	39928	50.3	21350	41.7	17508	41.4	11337	42.3	9652	43.6	1782	41.2	1194	46.2
County Percent of households with broadband connections with downstream speed ≥10mbps[6,*]																				
0-40%	66291	12.3	6265	21.6	501	17.7	9949	12.5	7247	14.2	3350	7.9	2156	8.0	1460	6.6	250	5.8	208	8.0
40.1-60%	148149	27.5	10306	35.5	918	32.4	20457	25.8	12838	25.1	9807	23.2	6428	24.0	4791	21.7	848	19.6	579	22.4
60.1-80%	283478	52.6	11457	39.4	1304	46.1	42225	53.2	27610	54.0	23614	55.8	14761	55.1	12722	57.5	2409	55.7	1427	55.2
80.1-100%	40986	7.6	1032	3.6	106	3.7	6727	8.5	3481	6.8	5533	13.1	3452	12.9	3142	14.2	815	18.9	373	14.4
Metropolitan Status[7, *]																				
Metropolitan	439494	81.6	20479	70.5	2015	71.2	69073	87.0	40931	80.0	33769	79.8	21514	80.3	18553	83.9	3706	85.7	2127	82.2
Non-metropolitan urban	89153	16.5	7695	26.5	717	25.3	9356	11.8	9306	18.2	7665	18.1	4737	17.7	3206	14.5	566	13.1	414	16.0
Rural	10257	1.9	886	3.0	97	3.4	929	1.2	939	1.8	870	2.1	546	2.0	356	1.6	50	1.2	46	1.8

Notes: Data come from the TAF files (2016-2020) for 41 states and Washington D.C. The analytic sample include n=799,452 children ages 3 to 17 with an index depression diagnosis. For children with multiple depression episodes identified, we only included their first episode.

[*]The distribution of this variable across settings within each year strata is significant at the p<0.001 level. Chi-Square tests were used to generate p-values.

[1] Applicants are asked to self-report their race and ethnicity; there is, however, variation in the data collection procedures across state Medicaid/CHIP programs. CMS provided technical guidance to states on submitting race/ethnicity data to the T-MSIS system. https://www.kff.org/medicaid/issue-brief/medicaid-administrative-data-challenges-with-race-ethnicity-and-other-demographic-variables/

[2] Eligibility information extracted in the month of episode initiation.

[3] Plan type information extracted in the month of episode initiation.

[4] Information extracted from 2020 and 2021 Area Health Resources Files. Year-specific county-level measures were linked for individuals identified in 2016-2020. Data available from: https://data.hrsa.gov/topics/health-workforce/ahrf

[5] Social deprivation index (SDI) generated based on American Community Survey data. Year-specific SDIs were available from 2016-2019. Year-specific SDI was linked for individuals identified in 2016-2019. SDI from 2019 was used to link with individuals identified in 2020. Data available from: https://www.graham-center.org/maps-data-tools/social-deprivation-index.html

[6] Broadband information available from 2016-2019. Year-specific broadband information was linked for individuals identified in 2016-2019. Broadband information from 2019 was used to link with individuals identified in 2020. Data available from: https://www.fcc.gov/general/fcc-form-477-additional-data

[7] Defined based on 2013 Rural-Urban Continuum Codes. Metro includes codes 1, 2, 3; Non-metro Urban includes codes 4, 5, 6, 7; Rural includes code 8, 9. For detailed documentation for each code, see: https://www.ers.usda.gov/data-products/rural-urban-continuum-codes/documentation/

© 2025 Cummings J et al. *JAMA Network Open*.

eTable 5. Adjusted Differences in the Receipt of Mental Health Visits and Pharmacotherapy among Publicly Insured Children with Index Depression Diagnosis

	Any Mental Health Visit (yes/no)		At least 4 Mental Health Visits (yes/no)		Any Pharmacotherapy (yes/no)		Minimally Adequate Pharmacotherapy (yes/no)	
	ME[1]	95% CI	ME[1]	95% CI	ME[1]	95% CI	ME[1]	95% CI
Mental Health Care Setting: 2016-2019								
Clinic only services	Ref (Pred Prob=66.4%)		Ref (Pred Prob=28.1%)		Ref (Pred Prob=31.8%)		Ref (Pred Prob=14.1%)	
Some (but ≤50%) telehealth services	13.61	(11.11, 16.11)	7.60	(5.80, 9.41)	11.72	(9.54, 13.90)	5.18	(4.18, 6.18)
Majority telehealth services	-7.45	(-10.97, -3.92)	-18.90	(-20.82, -16.99)	9.96	(5.39, 14.54)	7.78	(5.11, 10.46)
Any in-home services (no telehealth)	7.38	(2.91, 11.85)	12.66	(8.99, 16.33)	-5.14	(-6.20, -4.08)	-2.74	(-3.35, -2.13)
Any in-school svcs (no in-home, no telehealth)	1.74	(-2.78, 6.26)	7.40	(3.93, 10.87)	-11.75	(-12.98, -10.52)	-6.32	(-6.93, -5.71)
Mental Health Care Settings: 2020								
Clinic only services	Ref (Pred Prob=59.5%)		Ref (Pred Prob=21.2%)		Ref (Pred Prob=28.9%)		Ref (Pred Prob=13.3%)	
Some (but ≤50%) telehealth services	14.32	(11.57, 17.07)	15.28	(13.76, 16.79)	6.54	(5.70, 7.37)	4.84	(4.18, 5.49)
Majority telehealth services	17.79	(15.37, 20.22)	22.65	(21.03, 24.26)	-0.92	(-1.81, -0.02)	0.69	(0.06, 1.31)
Any in-home services (no telehealth)	10.16	(6.48, 13.84)	11.64	(8.85, 14.43)	-6.18	(-7.94, -4.43)	-3.37	(-4.57, -2.17)
Any in-school svcs (no in-home, no telehealth)	7.77	(0.23, 15.32)	8.18	(4.52, 11.84)	-14.36	(-16.36, -12.37)	-8.08	(-9.12, -7.03)
Age groups								
3-11 years	Ref		Ref		Ref		Ref	
12-17 years	0.42	(-0.55, 1.39)	-2.34	(-3.06, -1.62)	20.12	(19.69, 20.54)	9.01	(8.74, 9.28)
Sex								
Male	Ref		Ref		Ref		Ref	
Female	1.06	(0.76, 1.37)	1.24	(0.97, 1.51)	5.36	(5.05, 5.67)	2.71	(2.52, 2.90)
Race/Ethnicity[2]								
Non-Hispanic White	Ref		Ref		Ref		Ref	
Hispanic	0.12	(-1.82, 2.07)	-0.39	(-1.83, 1.06)	-5.75	(-6.49, -5.01)	-5.39	(-5.83, -4.95)
Non-Hispanic Amer.Indian/Alaskan Native	-7.25	(-10.03, -4.48)	-4.92	(-6.68, -3.17)	-3.96	(-5.22, -2.71)	-4.81	(-5.56, -4.06)
Non-Hispanic Asian / Pacific Islander	1.72	(-0.95, 4.39)	0.75	(-1.46, 2.95)	-2.82	(-4.32, -1.32)	-2.87	(-3.83, -1.90)
Non-Hispanic Black	0.17	(-0.85, 1.20)	-2.11	(-3.03, -1.20)	-8.56	(-9.07, -8.05)	-7.35	(-7.70, -7.00)
Non-Hispanic multi-racial group/Unknown	0.38	(-0.26, 1.02)	-0.46	(-1.02, 0.09)	-3.74	(-4.35, -3.13)	-3.47	(-3.90, -3.04)
Medicaid Eligibility Type[3]								
Low-income	Ref		Ref		Ref		Ref	
Disability	-8.01	(-9.26, -6.77)	-7.67	(-8.52, -6.83)	-2.67	(-3.44, -1.90)	-2.22	(-2.66, -1.79)
Other/Unknown	-2.27	(-3.27, -1.28)	-1.93	(-2.73, -1.12)	0.98	(0.14, 1.81)	0.10	(-0.53, 0.72)
Plan Type[4]								
Comprehensive managed care organization	Ref		Ref		Ref		Ref	
Primary care case management	-4.09	(-5.97, -2.22)	-5.47	(-7.32, -3.62)	1.45	(0.23, 2.68)	-0.17	(-1.00, 0.66)
Prepaid health plan	-2.64	(-4.04, -1.23)	-0.09	(-1.13, 0.95)	-0.73	(-1.42, -0.04)	-0.63	(-1.12, -0.14)
Other/Unknown	-8.89	(-10.51, -7.27)	-6.54	(-7.87, -5.20)	-0.67	(-1.33, 0.00)	-0.48	(-0.94, -0.01)
Attention deficit hyperactivity disorder (yes/no)	-2.33	(-3.71, -0.95)	-2.78	(-3.59, -1.96)	4.01	(3.06, 4.96)	3.31	(2.84, 3.77)
Anxiety disorder (yes/no)	7.75	(5.90, 9.61)	5.44	(4.42, 6.46)	18.96	(18.42, 19.50)	11.52	(11.12, 11.92)
Autism spectrum disorder (yes/no)	0.65	(-1.63, 2.92)	-0.38	(-1.78, 1.02)	1.73	(0.50, 2.95)	2.56	(1.73, 3.39)
Disruptive, impulse-control, & conduct disorders (yes/no)	3.77	(2.47, 5.07)	1.28	(0.12, 2.43)	2.64	(1.98, 3.29)	0.21	(-0.23, 0.65)
Trauma & other stressor-related disorders (yes/no)	8.54	(7.56, 9.52)	8.03	(7.25, 8.82)	10.18	(9.70, 10.66)	5.46	(5.12, 5.80)
Other mental health conditions (yes/no)	3.81	(2.44, 5.19)	1.69	(0.70, 2.69)	10.41	(9.36, 11.46)	4.62	(3.99, 5.24)

© 2025 Cummings J et al. *JAMA Network Open*.

eTable 5 (Cont.). Adjusted Differences in the Receipt of Mental Health Visits and Pharmacotherapy among Publicly Insured Children with Index Depression Diagnosis

	**Any Mental Health Visit (yes/no)**		**At least 4 Mental Health Visits (yes/no)**		**Any Pharmacotherapy (yes/no)**		**Minimally Adequate Pharmacotherapy (yes/no)**	
	ME[1]	95% CI	ME[1]	95% CI	ME[1]	95% CI	ME[1]	95% CI
County Non-Hispanic Black Population[5], Quartiles								
Q1	Ref		Ref		Ref		Ref	
Q2	-2.45	(-5.30, 0.40)	-0.02	(-1.99, 1.94)	1.35	(0.24, 2.47)	0.69	(0.05, 1.32)
Q3	-2.63	(-5.81, 0.55)	-1.55	(-3.68, 0.58)	2.28	(1.02, 3.54)	1.12	(0.39, 1.84)
Q4	-4.34	(-7.97, -0.72)	-2.55	(-5.12, 0.02)	1.71	(0.30, 3.12)	0.47	(-0.35, 1.29)
County Percent Hispanic Population[5], Quartiles								
Q1	Ref		Ref		Ref		Ref	
Q2	-0.80	(-2.54, 0.94)	-0.12	(-1.66, 1.42)	-0.72	(-1.63, 0.18)	-0.55	(-1.14, 0.04)
Q3	0.44	(-1.51, 2.39)	0.92	(-0.84, 2.68)	-1.96	(-3.06, -0.85)	-1.45	(-2.13, -0.76)
Q4	2.93	(0.29, 5.56)	2.25	(0.09, 4.41)	-4.69	(-6.12, -3.26)	-3.08	(-3.94, -2.21)
Social Deprivation Index[6], Quartiles								
Q1	Ref		Ref		Ref		Ref	
Q2	-0.71	(-3.26, 1.83)	-0.04	(-1.48, 1.40)	-2.08	(-3.04, -1.12)	-1.96	(-2.87, -1.06)
Q3	1.14	(-1.51, 3.79)	0.46	(-1.31, 2.23)	-2.24	(-3.34, -1.14)	-3.13	(-4.11, -2.16)
Q4	-0.13	(-3.16, 2.90)	-0.52	(-2.66, 1.63)	-4.47	(-5.79, -3.15)	-4.29	(-5.49, -3.08)
County Percent of households with broadband connections with downstream speed ≥10mbps[6,*]								
0-40%	Ref		Ref		Ref		Ref	
40.1-60%	1.70	(0.46, 2.94)	0.65	(-0.31, 1.61)	-0.56	(-1.24, 0.11)	-0.42	(-0.85, 0.01)
60.1-80%	1.90	(-0.28, 4.08)	1.19	(-0.36, 2.74)	-2.18	(-3.18, -1.18)	-1.01	(-1.62, -0.40)
80.1-100%	4.91	(1.25, 8.56)	2.94	(0.22, 5.66)	-3.68	(-5.34, -2.03)	-1.93	(-2.85, -1.02)
County-level Metropolitan Status[8]								
Metro	Ref		Ref		Ref		Ref	
Non-metro urban	-2.87	(-4.30, -1.45)	-1.75	(-2.82, -0.68)	1.66	(0.90, 2.42)	1.02	(0.57, 1.47)
Rural	-3.35	(-5.73, -0.97)	-2.41	(-4.09, -0.73)	-0.32	(-1.51, 0.86)	0.09	(-0.66, 0.84)

Notes: Data come from the TAF files (2016-2020) for 41 states and Washington D.C. The analytic sample include n=799,452 children ages 3 to 17 with an index depression diagnosis. For children with multiple depression episodes identified, we only included their first episode.

[1]ME are the marginal effects, which represent the adjusted percentage point change in each outcome variable associated with the setting of interest. These were estimated using margins MACRO following logistic regression models. Regression models controlled for child-level and county-level covariates listed in Table 1 and included state fixed effects, year indicators, and an interaction term between year and health care setting. The 95% confidence intervals are presented.

[2]Applicants are asked to self-report their race and ethnicity; there is, however, variation in the data collection procedures across state Medicaid/CHIP programs. CMS provided technical guidance to states on submitting race/ethnicity data to the T-MSIS system. https://www.kff.org/medicaid/issue-brief/medicaid-administrative-data-challenges-with-race-ethnicity-and-other-demographic-variables/

[3] Eligibility information extracted in the month of episode initiation.

[4] Plan type information extracted in the month of episode initiation.

[5] Information extracted from 2020 and 2021 Area Health Resources Files. Year-specific county-level measures were linked for individuals identified in 2016-2020. Data available from: https://data.hrsa.gov/topics/health-workforce/ahrf

[6] Social deprivation index (SDI) generated based on American Community Survey data. Year-specific SDIs were available from 2016-2019. Year-specific SDI was linked for individuals identified in 2016-2019. SDI from 2019 was used to link with individuals identified in 2020. Data available from: https://www.graham-center.org/maps-data-tools/social-deprivation-index.html

[7] Broadband information available from 2016-2019. Year-specific broadband information was linked for individuals identified in 2016-2019. Broadband information from 2019 was used to link with individuals identified in 2020. Data available from: https://www.fcc.gov/general/fcc-form-477-additional-data

[8] Defined based on 2013 Rural-Urban Continuum Codes. Metro includes codes 1, 2, 3; Non-metro Urban includes codes 4, 5, 6, 7; Rural includes code 8, 9. For detailed documentation for each code, see: https://www.ers.usda.gov/data-products/rural-urban-continuum-codes/documentation/

© 2025 Cummings J et al. *JAMA Network Open*.

eTable 6. Adjusted Differences in Minimally Adequate Depression Treatment among Publicly Insured Children

	ME[1]	95% CI
Mental Health Care Setting: 2016-2019		
Clinic only services	Ref (Pred Prob=38.4%)	
Some (but ≤50%) telehealth services	8.62	(6.93, 10.31)
Majority telehealth services	-6.87	(-9.16, -4.58)
Any in-home services (no telehealth)	9.15	(5.58, 12.72)
Any in-school services (no in-home, no telehealth)	2.36	(-0.90, 5.62)
Mental Health Care Setting: 2020		
Clinic only services	Ref (Pred Prob=32.3%)	
Some (but ≤50%) telehealth services	16.07	(14.56, 17.59)
Majority telehealth services	19.84	(18.20, 21.47)
Any in-home services (no telehealth)	6.92	(4.24, 9.60)
Any in-school services (no in-home, no telehealth)	0.82	(-2.68, 4.32)
Age groups		
3-11 years	Ref	
12-17 years	3.25	(2.48, 4.01)
Sex		
Male	Ref	
Female	3.01	(2.69, 3.34)
Race/Ethnicity[2]		
Non-Hispanic White	Ref	
Hispanic	-4.88	(-6.32, -3.43)
Non-Hispanic Amer.Indian/Alaskan Native	-8.32	(-10.09, -6.55)
Non-Hispanic Asian / Pacific Islander	-2.02	(-4.27, 0.23)
Non-Hispanic Black	-6.91	(-7.92, -5.91)
Non-Hispanic multi-racial group/Unknown	-3.45	(-4.10, -2.80)
Medicaid eligibility type[3]		
Low-income	Ref	
Disabilities	-8.12	(-8.96, -7.28)
Other/Unknown	-1.45	(-2.34, -0.56)
Plan Type[4]		
Comprehensive Managed Care Organization	Ref	
Primary Care Case Management	-3.20	(-4.87, -1.54)
Prepaid Health Plan	-0.44	(-1.44, 0.56)
Other/Unknown	-5.63	(-6.77, -4.49)
Attention deficit hyperactivity disorder (yes/no)	-0.82	(-1.74, 0.10)
Anxiety disorder (yes/no)	12.46	(11.36, 13.56)
Autism spectrum disorder (yes/no)	0.95	(-0.58, 2.49)
Disruptive, impulse-control, and conduct disorders (yes/no)	0.63	(-0.49, 1.75)
Trauma and other stressor-related disorders (yes/no)	9.07	(8.29, 9.85)
Other mental health conditions (yes/no)	3.35	(2.29, 4.41)
County-level Percent Non-Hispanic Black Population[5], Quartiles		
Q1	Ref	
Q2	1.08	(-0.78, 2.94)
Q3	-0.17	(-2.23, 1.88)
Q4	-1.76	(-4.05, 0.53)
County-level Percent Hispanic Population[5], Quartiles		
Q1	Ref	
Q2	-0.59	(-2.04, 0.87)
Q3	-0.22	(-1.88, 1.44)
Q4	-0.60	(-2.69, 1.49)

© 2025 Cummings J et al. *JAMA Network Open*.

Table 6 (Cont.). Adjusted Differences in Minimally Adequate Depression Treatment among Publicly Insured Children

	ME[1]	95% CI
County-level Social Deprivation Index[6], Quartiles		
Q1	Ref	
Q2	-0.54	(-2.02, 0.94)
Q3	-0.59	(-2.40, 1.22)
Q4	-2.87	(-4.91, -0.83)
County percent of households with broadband connections with downstream speed ≥10mbps[7,]		
0-40%	Ref	
40.1-60%	0.42	(-0.52, 1.35)
60.1-80%	0.21	(-1.21, 1.63)
80.1-100%	0.87	(-1.83, 3.56)
County-level Metropolitan Status[8]		
Metropolitan	Ref	
Non-metropolitan urban	-0.65	(-1.66, 0.36)
Rural	-2.08	(-3.69, -0.48)

Notes: Data come from the TAF files (2016-2020) for 41 states and Washington D.C. The analytic sample include n=799,452 children ages 3 to 17 with an index depression diagnosis. For children with multiple depression episodes identified, the sample only includes their first episode. The receipt of minimally adequate depression treatment is a dichotomous indicator for whether the child received ≥4 mental health visits in 12 weeks following index diagnosis or minimally adequate pharmacotherapy.

[1]ME are the marginal effects, which represent the adjusted percentage point change in each outcome variable associated with the setting of interest. These were estimated using margins MACRO following logistic regression models. Regression models controlled for child-level and county-level covariates listed in Table 1 and included state fixed effects, year indicators, and an interaction term between year and health care setting. The 95% confidence intervals are presented.

[2]Applicants are asked to self-report their race and ethnicity; there is, however, variation in the data collection procedures across state Medicaid/CHIP programs. CMS provided technical guidance to states on submitting race/ethnicity data to the T-MSIS system. https://www.kff.org/medicaid/issue-brief/medicaid-administrative-data-challenges-with-race-ethnicity-and-other-demographic-variables/

[3] Eligibility information extracted in the month of episode initiation.

[4] Plan type information extracted in the month of episode initiation.

[5] Information extracted from 2020 and 2021 Area Health Resources Files. Year-specific county-level measures were linked for individuals identified in 2016-2020. Data available from: https://data.hrsa.gov/topics/health-workforce/ahrf

[6] Social deprivation index (SDI) generated based on American Community Survey data. Year-specific SDIs were available from 2016-2019. Year-specific SDI was linked for individuals identified in 2016-2019. SDI from 2019 was used to link with individuals identified in 2020. Data available from: https://www.graham-center.org/maps-data-tools/social-deprivation-index.html

[7] Broadband information available from 2016-2019. Year-specific broadband information was linked for individuals identified in 2016-2019. Broadband information from 2019 was used to link with individuals identified in 2020. Data available from: https://www.fcc.gov/general/fcc-form-477-additional-data

[8] Defined based on 2013 Rural-Urban Continuum Codes. Metro includes codes 1, 2, 3; Non-metro Urban includes codes 4, 5, 6, 7; Rural includes code 8, 9. For detailed documentation for each code, see: https://www.ers.usda.gov/data-products/rural-urban-continuum-codes/documentation/

© 2025 Cummings J et al. *JAMA Network Open*.

eTable 7. Sensitivity Analysis Adjusted Differences in the Receipt of Mental Health Visits and Pharmacotherapy among Publicly Insured Children with Index Depression Diagnosis in All 50 States and Washington D.C.

	**Any Mental Health Visit (yes/no)**		**At least 4 Mental Health Visits (yes/no)**		**Any Pharmacotherapy (yes/no)**		**Minimally Adequate Pharmacotherapy (yes/no)**	
	ME[1]	95% CI	ME[1]	95% CI	ME[1]	95% CI	ME[1]	95% CI
Mental Health Care Setting: 2016-2019								
Clinic only services	Ref (Pred Prob=68.1%)		Ref (Pred Prob=29.0%)		Ref (Pred Prob=31.4%)		Ref (Pred Prob=13.8%)	
Some (but ≤50%) telehealth services	14.06	(11.91, 16.21)	9.70	(7.84, 11.56)	10.44	(8.70, 12.18)	4.40	(3.57, 5.23)
Majority telehealth services	-6.90	(-9.99, -3.82)	-17.60	(-19.57, -15.62)	9.13	(5.32, 12.94)	6.87	(4.62, 9.11)
Any in-home services (no telehealth)	6.49	(3.25, 9.73)	11.42	(8.63, 14.21)	-5.69	(-6.60, -4.77)	-3.01	(-3.53, -2.49)
Any in-school services (no in-home, no telehealth)	4.24	(-0.01, 8.50)	10.85	(7.69, 14.01)	-11.95	(-12.92, -10.98)	-6.28	(-6.77, -5.79)
Mental Health Care Setting: 2020								
Clinic only services	Ref (Pred Prob=61.6%)		Ref (Pred Prob=22.8%)		Ref (Pred Prob=28.9%)		Ref (Pred Prob=13.0%)	
Some (but ≤50%) telehealth services	14.47	(12.14, 16.79)	14.82	(13.50, 16.15)	6.12	(5.35, 6.89)	4.67	(4.09, 5.26)
Majority telehealth services	17.72	(15.70, 19.73)	21.89	(20.46, 23.32)	-0.87	(-1.67, -0.06)	0.83	(0.26, 1.39)
Any in-home services (no telehealth)	9.35	(6.41, 12.30)	10.43	(8.11, 12.75)	-6.70	(-8.29, -5.11)	-3.52	(-4.58, -2.46)
Any in-school services (no in-home, no telehealth)	9.88	(2.93, 16.82)	9.93	(6.58, 13.29)	-14.51	(-16.22, -12.81)	-7.87	(-8.78, -6.95)
Age groups								
3-5 years	Ref		Ref		Ref		Ref	
12-17 years	-0.17	(-1.02, 0.69)	-2.64	(-3.26, -2.02)	19.74	(19.38, 20.11)	8.72	(8.49, 8.95)
Sex								
Male	Ref		Ref		Ref		Ref	
Female	1.09	(0.83, 1.36)	1.24	(1.00, 1.48)	4.73	(4.43, 5.02)	2.29	(2.11, 2.46)
Race/Ethnicity[2]								
Non-Hispanic White	Ref		Ref		Ref		Ref	
Hispanic	0.79	(-0.91, 2.49)	0.04	(-1.25, 1.32)	-5.61	(-6.27, -4.95)	-5.10	(-5.50, -4.70)
Non-Hispanic Amer.Indian/Alaskan Native	-6.74	(-9.30, -4.18)	-4.94	(-6.48, -3.40)	-3.84	(-4.98, -2.69)	-4.65	(-5.32, -3.99)
Non-Hispanic Asian / Pacific Islander	1.84	(-0.52, 4.19)	0.85	(-1.04, 2.75)	-2.90	(-4.21, -1.59)	-2.70	(-3.53, -1.87)
Non-Hispanic Black	0.17	(-0.66, 1.00)	-1.68	(-2.38, -0.98)	-8.85	(-9.30, -8.40)	-7.23	(-7.53, -6.93)
Non-Hispanic multi-racial group/Unknown	0.57	(0.03, 1.12)	-0.35	(-0.84, 0.13)	-3.78	(-4.30, -3.26)	-3.42	(-3.79, -3.06)
Medicaid Eligibility Type[3]								
Low-income	Ref		Ref		Ref		Ref	
Disability	-7.32	(-8.33, -6.32)	-7.11	(-7.85, -6.37)	-2.66	(-3.30, -2.03)	-2.32	(-2.68, -1.96)
Other/Unknown	-2.48	(-3.47, -1.48)	-2.13	(-2.94, -1.32)	0.83	(0.05, 1.61)	-0.01	(-0.58, 0.57)
Plan Type[4]								
Comprehensive managed care organization	Ref		Ref		Ref		Ref	
Primary care case management	-3.80	(-5.38, -2.22)	-4.00	(-5.56, -2.45)	0.53	(-0.58, 1.64)	-0.35	(-1.13, 0.42)
Prepaid health plan	-2.90	(-4.15, -1.64)	0.27	(-0.68, 1.22)	-1.26	(-1.93, -0.60)	-0.77	(-1.23, -0.31)
Other/Unknown	-8.48	(-9.77, -7.20)	-5.32	(-6.35, -4.29)	-1.15	(-1.72, -0.57)	-0.96	(-1.37, -0.55)
Attention deficit hyperactivity disorder (yes/no)	-2.88	(-3.98, -1.78)	-3.17	(-3.86, -2.48)	4.50	(3.71, 5.30)	3.30	(2.91, 3.69)
Anxiety disorder (yes/no)	6.37	(4.77, 7.97)	4.97	(4.12, 5.82)	19.06	(18.57, 19.55)	11.29	(10.94, 11.64)
Autism spectrum disorder (yes/no)	-0.77	(-2.63, 1.08)	-0.68	(-1.92, 0.56)	2.15	(1.12, 3.18)	2.65	(1.94, 3.36)
Disruptive, impulse-control, and conduct disorders (yes/no)	2.18	(1.10, 3.26)	0.72	(-0.23, 1.68)	2.12	(1.56, 2.68)	-0.09	(-0.47, 0.28)
Trauma and other stressor-related disorders (yes/no)	7.10	(6.23, 7.97)	7.50	(6.76, 8.24)	9.94	(9.51, 10.38)	5.15	(4.85, 5.44)
Other mental health conditions (yes/no)	2.78	(1.55, 4.01)	1.01	(0.14, 1.88)	9.86	(8.99, 10.73)	4.28	(3.76, 4.79)

© 2025 Cummings J et al. *JAMA Network Open*.

eTable 7 (Cont.): Sensitivity Analysis - Adjusted Differences in the Receipt of Mental Health Visits and Pharmacotherapy among Publicly Insured Children with Index Depression Diagnosis in All 50 States and Washington D.C.

	**Any Mental Health Visit (yes/no)**		**At least 4 Mental Health Visits (yes/no)**		**Any Pharmacotherapy (yes/no)**		**Minimally Adequate Pharmacotherapy (yes/no)**	
	ME[1]	95% CI	ME[1]	95% CI	ME[1]	95% CI	ME[1]	95% CI
County Non-Hispanic Black Population[5], Quartiles								
Q1	Ref		Ref		Ref		Ref	
Q2	-1.81	(-4.43, 0.80)	0.10	(-1.66, 1.85)	1.11	(0.07, 2.14)	0.54	(-0.05, 1.12)
Q3	-2.06	(-5.09, 0.98)	-1.55	(-3.55, 0.45)	1.87	(0.67, 3.06)	0.81	(0.13, 1.49)
Q4	-3.17	(-6.48, 0.14)	-2.21	(-4.52, 0.11)	0.98	(-0.35, 2.31)	0.11	(-0.65, 0.86)
County Percent Hispanic Population[5], Quartiles								
Q1	Ref		Ref		Ref		Ref	
Q2	-0.32	(-1.80, 1.16)	0.25	(-0.99, 1.50)	-0.13	(-0.98, 0.73)	-0.18	(-0.71, 0.35)
Q3	0.41	(-1.24, 2.06)	0.95	(-0.44, 2.33)	-1.44	(-2.41, -0.48)	-1.12	(-1.71, -0.53)
Q4	2.64	(0.40, 4.89)	2.31	(0.54, 4.07)	-3.94	(-5.19, -2.68)	-2.52	(-3.28, -1.77)
Social Deprivation Index[6], Quartiles								
Q1	Ref		Ref		Ref		Ref	
Q2	-0.31	(-2.39, 1.77)	0.23	(-0.98, 1.44)	-1.71	(-2.51, -0.90)	-0.32	(-0.69, 0.06)
Q3	0.99	(-1.24, 3.22)	0.58	(-0.94, 2.11)	-1.85	(-2.79, -0.91)	-0.78	(-1.31, -0.24)
Q4	0.15	(-2.36, 2.65)	-0.17	(-2.01, 1.67)	-3.70	(-4.85, -2.55)	-1.57	(-2.37, -0.76)
County percent of households with broadband connections with downstream speed ≥10mbps[7]								
0-400 connections	Ref		Ref		Ref		Ref	
401-600 connections	1.73	(0.62, 2.84)	0.53	(-0.34, 1.40)	-0.56	(-1.16, 0.05)	-1.05	(-1.52, -0.57)
601-800 connections	2.07	(0.21, 3.93)	2.11	(0.75, 3.46)	-1.93	(-2.82, -1.04)	-1.43	(-1.97, -0.90)
801-1000 connections	4.56	(1.32, 7.80)	3.10	(0.70, 5.51)	-3.29	(-4.77, -1.82)	-2.67	(-3.31, -2.03)
County-level Metropolitan Status[8]								
Metro	Ref		Ref		Ref		Ref	
Non-metro urban	-2.35	(-3.59, -1.10)	-1.61	(-2.56, -0.66)	1.74	(1.07, 2.40)	1.09	(0.69, 1.50)
Rural	-2.86	(-5.02, -0.70)	-2.41	(-3.92, -0.89)	0.06	(-1.02, 1.14)	0.39	(-0.29, 1.07)

Notes: Data come from the TAF files (2016-2020) for 50 states and Washington D.C. The analytic sample includes n=994,974 children ages 3 to 17 with an index depression diagnosis. For children with multiple depression episodes identified, the sample only includes their first episode.

[1]ME are the marginal effects, which represent the adjusted percentage point change in each outcome variable associated with the setting of interest. These were estimated using margins MACRO following logistic regression models. Regression models controlled for child-level and county-level covariates listed in Table 1 and included state fixed effects, year indicators, and an interaction term between year and health care setting. The 95% confidence intervals are presented.

[2]Applicants are asked to self-report their race and ethnicity; there is, however, variation in the data collection procedures across state Medicaid/CHIP programs. CMS provided technical guidance to states on submitting race/ethnicity data to the T-MSIS system. https://www.kff.org/medicaid/issue-brief/medicaid-administrative-data-challenges-with-race-ethnicity-and-other-demographic-variables/

[3] Eligibility information extracted in the month of episode initiation.

[4] Plan type information extracted in the month of episode initiation.

[5] Information extracted from 2020 and 2021 Area Health Resources Files. Year-specific county-level measures were linked for individuals identified in 2016-2020. Data available from: https://data.hrsa.gov/topics/health-workforce/ahrf

[6] Social deprivation index (SDI) generated based on American Community Survey data. Year-specific SDIs were available from 2016-2019. Year-specific SDI was linked for individuals identified in 2016-2019. SDI from 2019 was used to link with individuals identified in 2020. Data available from: https://www.graham-center.org/maps-data-tools/social-deprivation-index.html

[7] Broadband information available from 2016-2019. Year-specific broadband information was linked for individuals identified in 2016-2019. Broadband information from 2019 was used to link with individuals identified in 2020. Data available from: https://www.fcc.gov/general/fcc-form-477-additional-data

[8] Defined based on 2013 Rural-Urban Continuum Codes. Metro includes codes 1, 2, 3; Non-metro Urban includes codes 4, 5, 6, 7; Rural includes code 8, 9. For detailed documentation for each code, see: https://www.ers.usda.gov/data-products/rural-urban-continuum-codes/documentation/

© 2025 Cummings J et al. *JAMA Network Open*.

eTable 8. Sensitivity Analysis - Adjusted Differences in Minimally Adequate Depression among Publicly Insured Children in All 50 States and Washington D.C.

	ME[1]	95% CI
Mental Health Care Setting: 2016-2019		
Clinic only services	Ref (Pred Prob=39.0%)	
Some (but ≤50%) telehealth services	9.77	(8.12, 11.42)
Majority telehealth services	-7.09	(-9.16, -5.01)
Any in-home services (no telehealth)	7.74	(5.05, 10.42)
Any in-school services (no in-home, no telehealth)	5.86	(2.77, 8.95)
Mental Health Care Setting: 2020		
Clinic only services	Ref (Pred Prob=33.5%)	
Some (but ≤50%) telehealth services	15.69	(14.38, 17.00)
Majority telehealth services	19.23	(17.82, 20.64)
Any in-home services (no telehealth)	5.84	(3.60, 8.08)
Any in-school services (no in-home, no telehealth)	2.91	(-0.39, 6.21)
Age groups		
3-5 years	Ref	
12-17 years	2.70	(2.03, 3.38)
Sex		
Male	Ref	
Female	2.70	(2.42, 2.98)
Race/Ethnicity[3]		
Non-Hispanic White	Ref	
Hispanic	-4.23	(-5.53, -2.94)
Non-Hispanic Amer.Indian/Alaskan Native	-8.21	(-9.78, -6.64)
Non-Hispanic Asian / Pacific Islander	-1.83	(-3.79, 0.13)
Non-Hispanic Black	-6.19	(-6.98, -5.41)
Non-Hispanic multi-racial group/Unknown	-3.18	(-3.73, -2.63)
Medicaid eligibility type[4]		
Low-income	Ref	
Disabilities	-7.64	(-8.38, -6.89)
Other/Unknown	-1.70	(-2.57, -0.83)
Plan Type[5]		
Comprehensive Managed Care Organization	Ref	
Primary Care Case Management	-2.68	(-4.10, -1.27)
Prepaid Health Plan	-0.36	(-1.30, 0.57)
Other/Unknown	-5.24	(-6.12, -4.36)
Attention deficit hyperactivity disorder (yes/no)	-1.24	(-2.03, -0.46)
Anxiety disorder (yes/no)	11.78	(10.84, 12.71)
Autism spectrum disorder (yes/no)	0.66	(-0.68, 1.99)
Disruptive, impulse-control, and conduct disorders (yes/no)	-0.07	(-0.98, 0.85)
Trauma and other stressor-related disorders (yes/no)	8.41	(7.70, 9.13)
Other mental health conditions (yes/no)	2.50	(1.55, 3.45)
County-level Percent Non-Hispanic Black Population[6], Quartiles		
Q1	Ref	
Q2	0.96	(-0.70, 2.61)
Q3	-0.57	(-2.48, 1.34)
Q4	-1.71	(-3.74, 0.33)
County-level Percent Hispanic Population[6], Quartiles		
Q1	Ref	
Q2	0.09	(-1.12, 1.30)
Q3	0.15	(-1.24, 1.53)
Q4	0.05	(-1.70, 1.80)

© 2025 Cummings J et al. *JAMA Network Open*.

eTable 8 (Cont.). Sensitivity Analysis - Adjusted Differences in Minimally Adequate Depression Treatment among Publicly Insured Children in All 50 States and Washington D.C.[1]

	ME[2]	95% CI
County-level Social Deprivation Index[6], Quartiles		
Q1	Ref	
Q2	-0.10	(-1.37, 1.17)
Q3	-0.33	(-1.90, 1.23)
Q4	-2.20	(-3.98, -0.42)
County percent of households with broadband connections with downstream speed ≥10mbps[7]		
0-400 connections	Ref	
401-600 connections	0.26	(-0.59, 1.11)
601-800 connections	1.14	(-0.13, 2.41)
801-1000 connections	1.12	(-1.31, 3.56)
County-level Metropolitan Status[8]		
Metropolitan	Ref	
Non-metropolitan urban	-0.51	(-1.41, 0.39)
Rural	-1.96	(-3.41, -0.51)

Notes: [1]Data come from the TAF files (2016-2020) for 50 states and Washington D.C. The analytic sample includes n=994,974 children ages 3 to 17 with an index depression diagnosis. For children with multiple depression episodes identified, the sample only includes their first episode. The receipt of minimally adequate depression treatment is a dichotomous indicator for whether the child received ≥4 mental health visits in 12 weeks following index diagnosis or minimally adequate pharmacotherapy.

[2]ME are the marginal effects, which represent the adjusted percentage point change in each outcome variable associated with the setting of interest. These were estimated using margins MACRO following logistic regression models. Regression models controlled for child-level and county-level covariates listed in Table 1 and included state fixed effects, year indicators, and an interaction term between year and health care setting. The 95% confidence intervals are presented.

[3]Applicants are asked to self-report their race and ethnicity; there is, however, variation in the data collection procedures across state Medicaid/CHIP programs. CMS provided technical guidance to states on submitting race/ethnicity data to the T-MSIS system. https://www.kff.org/medicaid/issue-brief/medicaid-administrative-data-challenges-with-race-ethnicity-and-other-demographic-variables/

[4] Eligibility information extracted in the month of episode initiation.

[4] Plan type information extracted in the month of episode initiation.

[5] Information extracted from 2020 and 2021 Area Health Resources Files. Year-specific county-level measures were linked for individuals identified in 2016-2020. Data available from: https://data.hrsa.gov/topics/health-workforce/ahrf

[6] Social deprivation index (SDI) generated based on American Community Survey data. Year-specific SDIs were available from 2016-2019. Year-specific SDI was linked for individuals identified in 2016-2019. SDI from 2019 was used to link with individuals identified in 2020. Data available from: https://www.graham-center.org/maps-data-tools/social-deprivation-index.html

[7] Broadband information available from 2016-2019. Year-specific broadband information was linked for individuals identified in 2016-2019. Broadband information from 2019 was used to link with individuals identified in 2020. Data available from: https://www.fcc.gov/general/fcc-form-477-additional-data

[8] Defined based on 2013 Rural-Urban Continuum Codes. Metro includes codes 1, 2, 3; Non-metro Urban includes codes 4, 5, 6, 7; Rural includes code 8, 9. For detailed documentation for each code, see: https://www.ers.usda.gov/data-products/rural-urban-continuum-codes/documentation/

© 2025 Cummings J et al. *JAMA Network Open*.

eFigure1. Sample Derivation Process

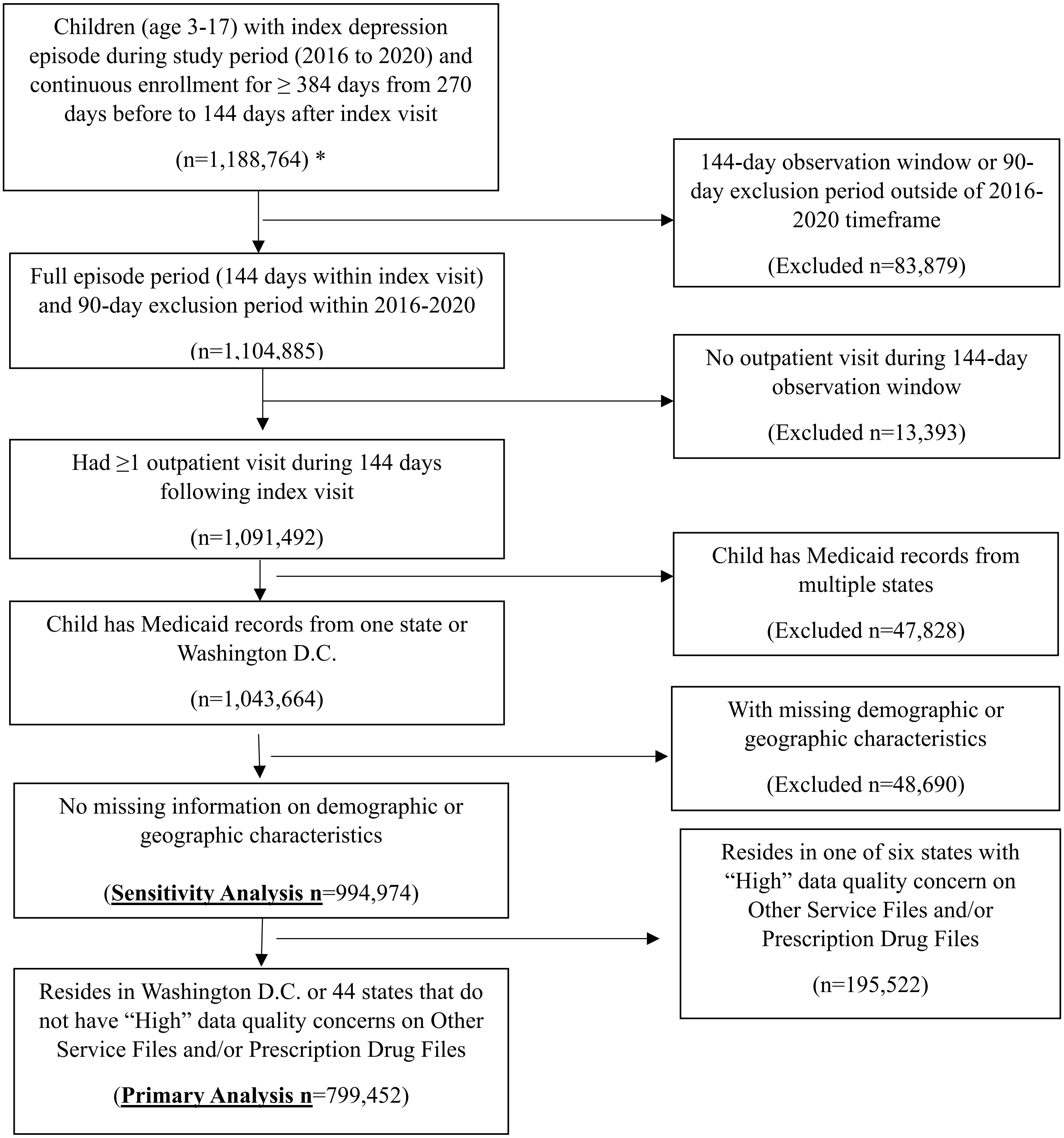

**Note: To identify the starting sample size, we first identified n=1,768,198 depression episodes during the study period, which included multiple episodes for the same child. We excluded 372,541 episodes because the beneficiary did not meet the criteria for continuous Medicaid enrollment before and after the depression episode. Of the remaining 1,395,657 episodes, we subsequently excluded n=206,893 episodes that were beyond the initial episode for a child. This derivation process yielded a starting sample size of n=1,188,764 children (age 3-17) with an index depression diagnosis during study period (2016 to 2020) and continuous enrollment for ≥ 384 days from 270 days before to 144 days after index visit.*

© 2025 Cummings J et al. *JAMA Network Open*.
