# Supplementary material for: Health Care Setting and Minimally Adequate Depression Treatment Among Publicly Insured Children
Source: JAMA Netw Open. 2025 Aug 28;8(8):e2528345. doi: 10.1001/jamanetworkopen.2025.28345 (PMC12395313; doi:10.1001/jamanetworkopen.2025.28345)
Supplement: Supplement 2. — Data Sharing Statement [file jamanetwopen-e2528345-s002.pdf]

Data Sharing Statement

Cummings. Health Care Setting and Minimally Adequate Depression Treatment Among Publicly Insured Children. *JAMA Netw Open*. Published August 28, 2025. doi:10.1001/jamanetworkopen.2025.28345

Data
Data available: No

Additional Information
Explanation for why data not available: The data cannot be shared per the terms of the data use agreement with the federal government.
